# 2020 World Society of Emergency Surgery updated guidelines for the diagnosis and treatment of acute calculus cholecystitis

**DOI:** 10.1186/s13017-020-00336-x

**Published:** 2020-11-05

**Authors:** Michele Pisano, Niccolò Allievi, Kurinchi Gurusamy, Giuseppe Borzellino, Stefania Cimbanassi, Djamila Boerna, Federico Coccolini, Andrea Tufo, Marcello Di Martino, Jeffrey Leung, Massimo Sartelli, Marco Ceresoli, Ronald V. Maier, Elia Poiasina, Nicola De Angelis, Stefano Magnone, Paola Fugazzola, Ciro Paolillo, Raul Coimbra, Salomone Di Saverio, Belinda De Simone, Dieter G. Weber, Boris E. Sakakushev, Alessandro Lucianetti, Andrew W. Kirkpatrick, Gustavo P. Fraga, Imitaz Wani, Walter L. Biffl, Osvaldo Chiara, Fikri Abu-Zidan, Ernest E. Moore, Ari Leppäniemi, Yoram Kluger, Fausto Catena, Luca Ansaloni

**Affiliations:** 1 General Surgery I, ASST Papa Giovanni XXIII Hospital, Bergamo, Italy; 2 Division of Surgery and Interventional Science, University College London, London, UK; 3 Department of Surgery, University of Verona, Verona, Italy; 4 General Surgery Trauma Team ASST-GOM Niguarda, Milan, Italy; 5 Department of Surgery, St. Antonius Ziekenhuis, Nieuwegein, Netherlands; 6 General Emergency and Trauma Surgery, Pisa University Hospital, Pisa, Italy; 7 HPB and Liver Transplant Surgery, Royal Free Hospital, London, UK; 8 HPB Surgeon Hospital Universitario La Princesa, Madrid, Spain; 9 Surgical Department, University of Macerata, Macerata, Italy; 10 Department of General and Emergency Surgery, University of Milano-Bicocca, Milan, Italy; 11 Department of Surgery, Harborview Medical Centre, University of Washington, Seattle, USA; 12 Unit of Digestive and HPB Surgery, CARE Department, Henri Mondor Hospital and University Paris-Est, Creteil, France; 13 General and Emergency Surgery, Bufalini Hospital, Cesena, Italy; 14 Emergency Room Brescia Spedali Civili General Hospital, Brescia, Italy; 15 Comparative Effectiveness and Clinical Outcomes Research Center-CECORC, Riverside University Health System Medical Center, Moreno Valley, CA USA; 16 Department of Surgery and Medicine, Insubria University, Varese, Italy; 17 Department of General Surgery, Azienda USL-IRCSS di Reggio Emilia, Guastalla Hospital, Guastalla, Italy; 18 Department of General Surgery Royal Perth Hospital, The University of Western Australia, Perth, Australia; 19 Research Institute at Medical University Plovdiv/University Hospital St George, Plovdiv, Bulgaria; 20 General, Acute Care, Abdominal Wall Reconstruction, and Trauma Surgery, Foothills Medical Centre, Calgary, AB Canada; 21 Division of Trauma Surgery, School of Medical Sciences, University of Campinas, Campinas, SP Brazil; 22 Department of Surgery, Sheri-Kashmir Institute of Medical Sciences, Srinagar, India; 23 Scripps Memorial Hospital La Jolla, La Jolla, CA USA; 24 Department of Surgery, College of Medicine, UAE University, Al Ain, UAE; 25 Ernest E Moore Shock Trauma Center at Denver Health, Denver, CO USA; 26 Abdominal Center Helsinki University Hospital, Helsinki, Finland; 27 Department of General Surgery, the Rambam Academic Hospital, Haifa, Israel; 28 Emergency Surgery, University Parma Hospital, Parma, Italy

**Keywords:** Acute cholecystitis, Early and delayed cholecystectomy, Surgery, Antibiotics, Gallbladder Drainage, High-risk patients, Guidelines

## Abstract

**Background:**

Acute calculus cholecystitis (ACC) has a high incidence in the general population. The presence of several areas of uncertainty, along with the availability of new evidence, prompted the current update of the 2016 WSES (World Society of Emergency Surgery) Guidelines on ACC.

**Materials and methods:**

The WSES president appointed four members as a scientific secretariat, four members as an organization committee and four members as a scientific committee, choosing them from the expert affiliates of WSES. Relevant key questions were constructed, and the task force produced drafts of each section based on the best scientific evidence from PubMed and EMBASE Library; recommendations were developed in order to answer these key questions. The quality of evidence and strength of recommendations were reviewed using the Grading of Recommendations Assessment, Development and Evaluation (GRADE) criteria (see https://www.gradeworkinggroup.org/). All the statements were presented, discussed and voted upon during the Consensus Conference at the 6th World Congress of the World Society of Emergency Surgery held in Nijmegen (NL) in May 2019. A revised version of the statements was voted upon via an online questionnaire until consensus was reached.

**Results:**

The pivotal role of surgery is confirmed, including in high-risk patients. When compared with the WSES 2016 guidelines, the role of gallbladder drainage is reduced, despite the considerable technical improvements available. Early laparoscopic cholecystectomy (ELC) should be the standard of care whenever possible, even in subgroups of patients who are considered fragile, such as the elderly; those with cardiac disease, renal disease and cirrhosis; or those who are generally at high risk for surgery. Subtotal cholecystectomy is safe and represents a valuable option in cases of difficult gallbladder removal.

**Conclusions, knowledge gaps and research recommendations:**

ELC has a central role in the management of patients with ACC. The value of surgical treatment for high-risk patients should lead to a distinction between high-risk patients and patients who are not suitable for surgery. Further evidence on the role of clinical judgement and the use of clinical scores as adjunctive tools to guide treatment of high-risk patients and patients who are not suitable for surgery is required. The development of local policies for safe laparoscopic cholecystectomy is recommended.

## Background

The estimated overall prevalence of gallstones is 10–15% in the general population, with some differences across countries. Between 20 and 40% of patients with gallstones will develop gallstone-related complications, with an incidence of 1–3% annually; acute calculus cholecystitis (ACC) is the first clinical presentation in 10–15% of the cases [1–6]. Cholecystectomy is the most common therapeutic approach for ACC and is considered the standard of care for gallstone disease for the majority of patients. However, considering the heterogeneity of clinical scenarios, the variability in hospital facilities and in the availability of expertise, the management of patients with right upper quadrant abdominal pain may vary.

In 2016, the World Society of Emergency Surgery (WSES) published the first edition of their guidelines for ACC [7], which presented different diagnostic and therapeutic algorithms, compared with the Tokyo Guidelines (TG), known at that time as Tokyo Guidelines 2013 (TG13) [8]. In particular, the direct link between diagnostic criteria for ACC, severity classification and therapeutic indications described in the TG13 are limited by lack of quality evidence. The approach of the WSES guidelines was to simplify the initial management of patients presenting with suspected ACC. The literature review, the discussion of the relevant evidence and the statements made during the consensus conference (CC) held in Jerusalem in 2015 (Third WSES International Congress) supported surgery as the gold standard treatment for all patients with ACC, with two exceptions: patients who refuse surgery, and patients for whom surgery would be considered as ‘very high risk’, although no clear consensus was reached on this second issue. Moreover, the 2016 WSES Guidelines on ACC included discussions on unclear areas, such as diagnosis, evaluation of the surgical risk and appropriate management of associated common bile duct stones (CBDS).

In 2017, the WSES joined the Italian Society for Geriatric Surgery during a CC regarding the management of ACC in the elderly, with the aim of investigating this subgroup of fragile patients, considered at ‘very high risk’ for surgery. There was lack of agreement supporting the surgical management of ACC in the elderly and considering old age as a contraindication for surgery by itself. The authors found substantial lack of high-quality studies on this topic [9].

The WSES, after evaluating the 2018 edition of the TG (TG18) on ACC [10], found that this new edition reached conclusions that were closer to the recommendations of the 2016 WSES guidelines on ACC, especially in terms of a more liberal indication for surgery including grade 3 ACC. However, some differences remain when comparing the WSES guidelines and the TG (all editions), as evident in the recommendations in the current updated guidelines. A combined event, WSES and TG group could be an opportunity to share experiences considering different perspectives.

Since the publication of the 2016 WSES Guidelines and the TG18, the management of the high-risk patients with ACC was investigated in a randomized controlled trial (RCT), known as the CHOCOLATE trial [11]. Loozen and collaborators compared cholecystectomy to percutaneous catheter drainage in high-risk surgical patients. This research group has joined with other experts in contributing to this edition of the WSES guidelines on ACC.

## Materials and methods

In 2018, the Scientific Board of the 6thWorld Congress of the WSES endorsed its president to organize a CC on ACC in order to update the previous WSES Guidelines. The WSES president appointed four members as a scientific secretariat, four members as an organization committee and four members as a scientific committee, choosing them from the expert affiliates of the WSES. Relevant key questions regarding the diagnosis and treatment of ACC were developed and divided into seven sections, in order to analyse the topic and update the guidelines with the currently available evidence. Under the supervision of the scientific secretariat, a bibliographic search related to these questions was performed, using electronic search of PubMed and EMBASE databases in May 2019, with no date or language restrictions. An additional manual search of the literature was performed by members of the working groups involved in the analysis of the papers and the development of the guidelines. The topics and sections, the key questions and the related key words used to develop the update on ACC are available in Table 1.

**Table 1:** Sections/topics, key questions and key words

Section/topic	Key questions	Key words
1. Diagnosis of Acute Calculus Cholecystitis	Which is the most reliable approach for the diagnosis of ACC? Which initial imaging technique should be used in case of a suspected diagnosis of ACC? Which is the role of other imaging techniques (e.g. Hepatobiliary iminodiacetic acid—HIDA scan, abdominal computed tomography—CT scan and magnetic resonance) in the diagnosis of ACC?	Acute calculus cholecystitis Diagnosis, Ultrasound, Gallstones disease diagnosis
2. Associated common bile duct stones (CBDS)	Are elevated LFTs or bilirubin sufficient for the diagnosis of CBDS in patients with ACC? Which imaging features are predictive of CBDS in patients with ACC? Which tests should be performed to assess the risk of CBDS in patients with ACC? Which is the best tool to stratify the risk for CBDS in patients with ACC? Which actions are warranted in patients with ACC and at moderate for CBDS? Which actions are warranted in patients with ACC and at high risk for CBDS? Which is the appropriate treatment of CBDS in patients with ACC?	Common bile duct stone; choledocholithiasis; endoscopic ultrasound, MRCP, ERCP
3. Surgical treatment of acute calculus cholecystitis	Which is the preferred first line of treatment for patients with ACC? When should laparoscopic cholecystectomy be avoided in patients with ACC? Is laparoscopic cholecystectomy safe and feasible for patients with ACC who have liver cirrhosis, who are older than 80 years and who are pregnant? Which surgical strategies should be adopted in case of difficult anatomic identification during cholecystectomy for ACC? When should conversion from laparoscopic to open cholecystectomy be considered in patients with ACC?	Acute calculus cholecystitis, Surgery, Laparoscopy, Laparotomy, Cholecystectomy, Partial cholecystectomy, Subtotal cholecystectomy, Cirrhosis, Pregnancy
4. Timing of cholecystectomy in people with acute calculus cholecystitis	Which is the optimal timing for laparoscopic cholecystectomy in patients with ACC?	Acute calculus cholecystitis, acute cholecystitis
5.Risk prediction in patients with acute calculus cholecystitis	How can the prognosis and surgical risk be assessed for patients with ACC?	Acute calculus cholecystitis, Gallstone disease, Surgical risk score, High risk patient, old patient, PPossum score, Apache score
6. Alternative treatment for patients who are not suitable for surgery: non-operative management and gallbladder drainage techniques	When should Non-Operative Management (NOM) be considered for patients with ACC? Which is the first-choice treatment for ACC in high risk patients? Which is the role of gallbladder drainage in patients with ACC who are not suitable for surgery? Should delayed cholecystectomy be offered to patients with ACC after the reduction of perioperative risk? Can endoscopic gallbladder drainage be considered an alternative to PTGBD in patients with ACC who are not suitable for surgery? Which is the role of endoscopic transmural ultrasound-guided gallbladder drainage (EUS-GBD) in patients with ACC who are not suitable for surgery?	Gallstones Dissolution, No-surgery gallstones, Extra-corporeal shock wave lithotripsy, Acute calculus cholecystitis, Gallstone disease, Management Gallstones, Endoscopy, Gallstone removal, Observation cholecystitis, Non operative management cholecystitis, Gallbladder drainage Percutaneous gallbladder drainage, Cholecystostomy, High Risk Patient, Stent
7. Antibiotic treatment on acute calculus cholecystitis	Which is the optimal antibiotic treatment for patients with uncomplicated ACC? Which is the optimal antibiotic treatment for patients with complicated ACC? Which is the role of microbiological cultures and sensitivities in patients with ACC?	Antibiotics, Acute calculus cholecystitis, Gallstone disease, Management Gallstones

Before the CC, the statements and recommendations were reviewed by the representatives responsible for each of the sections, creating a draft version of the guidelines. The quality of evidence and strength of recommendations were reviewed using the Grading of Recommendations Assessment, Development and Evaluation (GRADE) criteria (see https://www.gradeworkinggroup.org/). Specifically, the quality of evidence was graded as ‘High’, ‘Moderate’, ‘Low’ or ‘Very low’ and the strength of a recommendation was indicated as either ‘Strong’ or ‘Weak’. Consensus had previously been defined as 70% or more of the votes being in agreement. During the 6th World Congress of the WSES held in Nijmegen, the Netherlands in May 2019, each question was discussed and voted upon by the audience (votes were either ‘agree’ or ‘disagree’). The percentage of agreement was recorded immediately; in case of disagreement, the statement was modified following discussion. After the CC, the president and representatives reviewed the guidelines in response to the comments and the revised version of the statements was voted upon via an online questionnaire until consensus was reached. Throughout the period of the elaboration of the current guidelines, repeated literature searches were carried out in order to maximize the inclusion of relevant evidence (last literature search: May 2020).

These Guidelines should be considered an adjunctive tool for decision making, but they are not a substitute for the surgeon’s judgement in specific clinical situations.

**Fig. 1:**
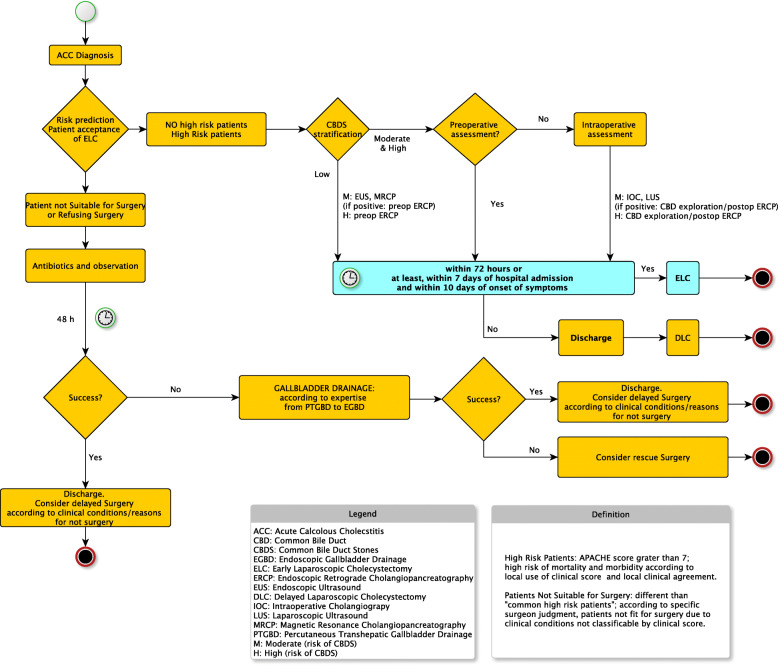
2020 WSES Flowchart for the management of patients with acute calcolus cholecystitis

In Appendix 1, the reader can find the summary of statements with short explanation of the supporting scientific evidence while details are in the body of the paper. Figure 1 represents the 2020 WSES flowchart for the management of ACC patients.

The WSES committee for guidelines development is responsible for the continuous evaluation of evidence available about acute cholecystitis. The present guidelines will be updated in case of significant changes based on new evidence.

## Section 1. Diagnosis of ACC

**1.1 As no feature has sufficient diagnostic power to establish or exclude the diagnosis of ACC, it is recommended not to rely on a single clinical or laboratory finding. #QoE: high; SoR: strong#**

**1.2 For the diagnosis of ACC, we suggest using a combination of detailed history, complete clinical examination, laboratory tests and imaging investigations. However, the best combination is not known. #QoE: very low; SoR: weak#**

Comment: useful features for the diagnosis of ACC are:

- History and clinical examination: fever, right upper quadrant pain or tenderness, vomiting or food intolerance; Murphy’s sign
- Laboratory tests: elevated C-reactive protein, elevated white blood cell count
- Imaging: signs suggestive of gallbladder inflammation

The recommendations of the 2016 WSES guidelines were mainly based on two studies: a systematic review and meta-analysis by Trowbridge et al. [12] and a prospective diagnostic study by Eskelinen et al. [13]. This evidence, although flawed by the limitations described below, remains relevant and the associated statement remains valid.

The paper by Trowbridge et al. [12] included 17 studies, which reported a quantitative assessment of history, physical examination and/or laboratory tests for the diagnosis of acute cholecystitis. The results showed that, with the exception of Murphy’s sign (positive likelihood ratio—LR 2.8; 95% CI 0.8–8.6) and right upper quadrant tenderness (negative LR0.4; 95% CI 0.2–1.1)—although the 95% confidence intervals included 1.0 in both cases, none of the clinical signs or laboratory tests showed LRs higher than 1.6 or negative LRs lower than 0.4. Limitations were identified in a possible selection bias, as patients with abdominal pain and patients with a suspected diagnosis of acute cholecystitis were included in the study, and in a heterogeneous definition of the diagnosis of acute cholecystitis.

The article by Eskelinen et al. [13] evaluated more than 1300 patients and revealed a good diagnostic yield with a combination of findings from history, physical examination and laboratory tests, reporting a positive LR of 25.7 and a negative LR of 0.24.

The TG criteria for the diagnosis of cholecystitis include clinical signs, laboratory tests and imaging features [14]. After the publication of the paper by Yokoe et al. in 2012 [15] reporting 91.2% sensitivity and 96.9% specificity, three studies reporting the validation of the TG diagnostic criteria were found. Although published in 2017, one study focused on the TG07 rather than the more recent TG13 [16]. A cross-sectional study [17] evaluated the validity of fever, inflammatory markers and US findings as a validation of the TG13 criteria. At multivariate analysis, only neutrophil count was statistically associated with the diagnosis of acute cholecystitis (*p* <0.0001), with a 70% sensitivity and 65.8% specificity. Overall, accuracy of the TG13 criteria was low at 60.3%. The TG13 correctly predicted 83.1% of all confirmed ACC, but over-diagnosed ACC in 62.5% of normal gallbladders. More than half of eligible patients did not undergo US and were excluded from the study; this represents a major source of potential selection bias. A cross-sectional study on the possible limitations of the TG 13 has reported a 53.4% sensitivity in diagnosing acute cholecystitis [18]. However, some uncertainty regarding the sample population and the lack of detail in sensitivity calculation indicates that the data should be interpreted with caution. The revision of the TG criteria performed in 2018 did not include a clinical evaluation of the diagnostic criteria [14]. Considering the heterogeneity of these findings, the reliability of the TG13 criteria for the diagnosis of ACC appears to be limited.

***Which initial imaging technique should be used in case of a suspected diagnosis of ACC?***

**1.3 We recommend the use of abdominal ultrasound (US) as the preferred initial imaging technique, in view of its cost-effectiveness, wide availability, reduced invasiveness and good accuracy for gallstones disease. #QoE: high; SoR: strong#**

Comment: abdominal US is a reliable investigation method; however, it may be of limited utility to rule in or rule out the diagnosis of ACC according to the adopted US criteria.

Neither meta-analysis nor studies with adequate quality of evidence have been published on this topic since the publication of the 2016 WSES guidelines.

In 2012, Kiewet et al. published a systematic review and meta-analysis [19] of diagnostic performance of different imaging techniques in ACC; abdominal ultrasound was not as accurate as it is for the diagnosis of gallstones. The meta-analysis was based on the results of 26 studies including a total of 2847 patients. The sensitivity in individual studies ranged from 50 to 100% and specificity from 33 to 100%. Summary sensitivity and specificity were 81% (95% CI 75 to 87%) and 83% (95% CI 74 to 89%), respectively. However, strong heterogeneity in the diagnostic performance of abdominal US was reported: the inconsistency index was 80% for sensitivity and 89% for specificity. Notwithstanding these limitations, the widespread availability, lack of invasiveness, lack of exposure to ionizing radiation and the reduced costs make abdominal US the preferred initial imaging technique in suspected ACC.

Published data from eight cross-sectional studies [20–27] confirmed the heterogeneity of diagnostic values, diagnostic index and standard reference for the final diagnosis of ACC. Traditional US presented wide ranges of sensitivity (from 26 to 100% [20–26]), specificity (from 62 to 88.1% [22–27]), positive predictive value (PPV) and negative predictive value (NPV 35% to 93.7% and 52% to 97.1%, respectively), as well as positive LR (1.29 to 4.7) and negative LR (0.16 to 0.93) [23, 24]. Global accuracy has been reported in two studies and varied from 70.1 to 79% [26, 27].

In one study, the absence of gallstones was used to rule out the diagnosis of acute cholecystitis in patients presenting to the emergency department for suspected cholecystitis [23]. Overall, the sensitivity of the simplified definition of a positive ultrasonography test was100%, as compared to the standard definition, i.e. the presence of gallstones and at least one of the ultrasonography signs of acute cholecystitis, which showed a sensitivity of 87% (95% CI 66–97%) and specificity of 82 (95% CI 74–88%); prevalence was less than 15%, NPV was 97% (95% CI 93–-99%) and PPV was low at 44% (95% CI 29–59%).

Considering the limits of abdominal US, one study has evaluated new ultrasonography criteria for the diagnosis of acute cholecystitis. Kim et al. [26] evaluated the added value of point shear-wave elastography (pSWE) in the diagnostic performance of conventional US for the diagnosis of ACC. Based on the assumption that transient increase in hepatic blood flow observed in case of acute cholecystitis increases liver stiffness, the authors proposed to use a measure of liver stiffness by pSWE and to evaluate its diagnostic yield for ACC in a two-observer analysis. Compared to conventional US, pSWE significantly increased diagnostic accuracy (area under the curve—AUC from 79 to 96.3% and from 77 to 96.2%, *p* < 0.001) and specificity (from 62 to 95%, *p* < 0.001). The difference in sensitivity was not significant, being 88 versus 92% (*p* = 0.45) in the US only group and 86–92% (*p* = 0.26) in the US plus pSWE group. Although the results appear promising, the technique requires expertise; moreover, 18.5% of patients were excluded due to a potential limitation of the technique, therefore reducing the external validity of the study.

Another study by Ra et al. [27] has reported the use of superb microvascular imaging (SMI) in the diagnosis of acute cholecystitis. The SMI technique is similar to Color-Doppler US and is used to detect the micro vasculature and slow flow of the liver, using a special filtering technique. The authors hypothesised that hyperaemic changes within the gallbladder bed of the liver, detected by SMI, and may be used for the diagnosis of acute cholecystitis. This inter-observer study on 54 patients showed a significant increase of the AUC from 72.9 to 85% (*p* = 0.02) with the use of SMI. The need for specific expertise, the small number of patients and the poor reference standard limit the significance of this study.

***What is the role of other imaging techniques (e.g. Hepatobiliary iminodiacetic acid - HIDA scan, Abdominal Computed Tomography - CT scan and Magnetic Resonance Imaging - MRI) in the diagnosis of ACC?***

**1.4 We suggest the use of further imaging for the diagnosis of ACC in selected patients, depending on local expertise and availability. Hepatobiliary iminodiacetic acid (HIDA) scan has the highest sensitivity and specificity for the diagnosis of ACC as compared to other imaging modalities. Diagnostic accuracy of computed tomography (CT) is poor. Magnetic resonance imaging (MRI) is as accurate as abdominal US. #QoE: moderate; SoR: strong#**

Comment: in clinical practice, HIDA scan utilisation is limited due to the required resources and time.

No study with a high level of evidence was published on this topic since the publication of the 2016 WSES guidelines.

A cross-sectional study [28] evaluated the incremental benefits of cystic duct enhancement detected by CT for the diagnosis of cholecystitis in patients without visibly impacted gallstones. When considering cystic duct enhancement, the accuracy and sensitivity of the diagnosis increased significantly, while no significant difference was reported for specificity. Interestingly, diagnostic accuracy increased for the less experienced radiologist, from 75.4 to 87.3% (*p* = 0.015). However, this case control study has some methodological flaws limiting its quality.

A study considering only patients with a definitive diagnosis of acute cholecystitis compared the diagnostic sensitivities of US, CT and HIDA scan [20]. The results confirmed the higher sensitivity of HIDA over US and CT with respective values of 84.2%, 67.3% and 59.8% (*p* = 0.017). No difference was found when comparing CT and US (*p* = 0.09).

A study comparing sensitivity of CT and US [21] showed different results, reporting higher sensitivity of CT, compared with US(92% vs. 79%, *p* = 0.015). In this study, a retrospective cohort of patients was added based on prospectively collected data from patients with a diagnosis of ACC confirmed by pathology or intra-operative findings. Indication for CT and timing between index and reference standards were not reported.

One study on a time-saving HIDA scan technique reported a high inter-rate agreement (Cohen’s kappa coefficient = 0.92) between the novel time-saving technique and the conventional examination [29]. Two other studies reported diagnostic values of HIDA scan as 86.7–89.3% for sensitivity and 66.8–79% for specificity [21, 22].

## Section 2. Associated common bile duct stones: which tools to use for suspicion and diagnosis at presentation?

Choledocholithiasis, i.e. the presence of common bile duct stones (CBDS), is reported to occur in 10% to 20% of gallstone cases, with lower incidence, ranging from 5 to 15 %, in case of ACC [30–33]. Investigations for CBDS require time and may delay surgical treatment. Due to the relatively low incidence of CBDS during ACC, the main issue is to select patients with a high likelihood of CBDS, who would benefit from further diagnostic tests and removal of CBDS. An uncommon condition that mimics CBDS is Mirizzi syndrome, which occurs in less than 1% of patients with gallstones. Preoperative investigations may help in the diagnosis, although the vast majority of cases are identified at surgery [34, 35].

The only new study on this topic was a cross-sectional study on the role of liver function tests (LFTs) [36]. The authors evaluated the role of LFTs and the role of early follow-up in the diagnosis of CBDS in ACC. The most reliable LFT was gamma-glutamyl transpeptidase (GGT), with a sensitivity of 80.6% and a specificity of 75.3%, using a cut-off level of 224 IU/L. PPV was 50%, while NPV was 91.4%. The results also showed a significant decrease of LFTs within the non-CBD groups at 4-day follow-up, which was not evident in the ACC + CBDS group—with the exception of alanine aminotransferase (ALT). Moreover, in the CBDS group, all LFTs values improved significantly after the removal of the CBDS at a mean follow-up time of 4.3 days.

One flaw of the study is that index diagnosis depends to some extent on the reference standard. Given the retrospective design of the study, it should be considered that the diagnosis of CBDS is assessed with endoscopic retrograde cholangio-pancreatography (ERCP), which is mainly prompted by the presence of elevated LFTs; this may represent a source of bias. No systematic intra-operative cholangiography (IOC) was performed.

***Are elevated LFTs or bilirubin sufficient for the diagnosis of CBDS in patients with ACC?***

**2.1 We recommend against the use of elevated LFTs or bilirubin as the only method to identify CBDS in patients with ACC, in which case we recommend performing further diagnostic tests. #QoE: moderate; SoR: strong#**

Historically, LTFs have played a major role in determining the presence of CBDS. However, the majority of published studies did not consider patients with ACC and included asymptomatic gallstones. Normal LFTs have a NPV of 97%, whereas the PPV of any abnormal LFTs is only 15% [37]. The elevation of LFTs is a poor tool for the prediction of CBDS, even in patients without ACC; the literature ranging from 25 to 50% [30, 38, 39]. In patients with ACC, LFTs may be altered due to the acute inflammatory process of the gallbladder and the biliary tree, rather than direct biliary obstruction; a proportion ranging between 15 and 50% of patients with ACC show elevation in LFTs without CBDS. Song et al. demonstrated that 424 out of 1178 patients with ACC had increased LFTs, namely ALT and aspartate transaminase (AST) greater than twice reference levels. Of these, only 246 (58%) had CBDS [40]. Chang et al. showed that 51% and 41% of patients with ACC without CBDS had elevated ALT and AST, respectively. However, increased bilirubin levels with leucocytosis may predict gangrenous cholecystitis [41]. Padda et al. found that approximately 30% of patients with ACC without CBDS had abnormal alkaline phosphatase (ALP) and/or bilirubin, and 50% had abnormal ALT. Among patients with ACC and CBDS, 77% had raised ALP, 60% had abnormal bilirubin and 90% had elevated ALT; multivariate analysis showed that increased common bile duct size and elevated ALT and ALP were predictors of CBDS [42]. The diagnostic accuracy increases for cholestasis tests, such as serum bilirubin, with the duration and the severity of obstruction. Specificity of serum bilirubin levels for CBDS was 60% with a cut-off level of 1.7 mg/day and 75% with a cut-off level of 4 mg/dl [38]; however, mean level of bilirubin in patients with CBDS is generally lower (1.5 to 1.9 mg/dl) [30, 39].

A recent meta-analysis reported the diagnostic accuracy of serum bilirubin and serum ALP at two cut-off values for each test. Serum bilirubin at a cut-off of 22.23 μmol/L had a sensitivity of 0.84 (95% CI 0.65 to 0.94) and a specificity of 0.91 (95% CI 0.86 to 0.94). Bilirubin at a cut-off of greater than twice the normal limit, had a sensitivity of 0.42 (95% CI 0.22 to 0.63) and a specificity of 0.97 (95% CI 0.95 to 0.99). For ALP at a cut-off of greater than 125 IU/L, sensitivity was 0.92 (95% CI 0.74 to 0.99) and specificity was 0.79 (95% CI 0.74 to 0.84). For ALP at a cut-off of greater than twice the normal limit, sensitivity was 0.38 (95% CI 0.19 to 0.59) and specificity was 0.97 (95% CI 0.95 to 0.99) [43].

***Which imaging features are predictive of CBDS in patients with ACC?***

**2.2 We suggest considering the visualization of a stone in the common bile duct at transabdominal US as a predictor of CBDS in patients with ACC. #QoE: very low; SoR: weak#**

**2.3 An increased diameter of common bile duct, an indirect sign of stone presence, is not sufficient to identify ACC patients with CBDS and we therefore recommend performing further diagnostic tests. #QoE: high; SoR: strong#**

Abdominal US is the preferred imaging technique for the diagnosis of ACC; the common bile duct can be visualized and investigated at the same time. A meta-analysis by Gurusamy et al. investigated the diagnostic potential of US [43]: sensitivity ranged from 0.32 to 1.00 with a summary sensitivity of 0.73 (95% CI 0.44 to 0.90), while specificity ranged from 0.77 to 0.97 with a summary specificity of 0.91 (95% CI 0.84 to 0.95).

In a retrospective analysis, Boys et al. [44] found that the mean common bile duct diameter seen at abdominal US in ACC patients without and with CBDS was 5.8 and 7.1 mm, respectively (*p* = 0.004). A CBD diameter larger than 10 mm was associated with a 39 % incidence of CBDS, while diameter smaller than 9.9 mm was associated with CBDS in 14%. The authors concluded that common bile duct diameter is not sufficient on its own to identify patients having significant risk for CBDS.

***Which tests should be performed to assess the risk of CBDS in patients with ACC?***

**2.4 In order to assess the risk for CBDS, we suggest performing liver function tests (LFTs), including ALT, AST, bilirubin, ALP, GGT and abdominal US in all patients with ACC. #QoE: low; SoR: weak#**

Several scores for the prediction of CBDS have been proposed and validated; however, none of the proposed scores is specific for ACC. The implementation of these predictive scores in clinical practice remains poor [38–40]; all scores consider different combinations of the same clinical variables. Barkun et al. [38] combined age > 55 years, elevated serum bilirubin, dilated common bile duct and evidence of CBDS; Menezes et al. [45] combined age > 55 years, male sex, ascending cholangitis, dilated common bile duct, CBDS and abnormal LFTs; Soltan et al. [46] included history of symptomatic disease, abnormal liver function tests, dilated common bile duct and presence of CBDS; Sun et al. [47] included male sex, abnormal liver function test and dilated common bile duct; Sarli et al. [48] combined positive AUS and abnormal liver function tests.

The American Society of Gastrointestinal Endoscopy and the Society of American of Gastrointestinal Endoscopic Surgeons combined the published validated clinical scores and proposed a risk stratification of CBDS in three different classes, defined as follows: low risk (< 10%), moderate risk (10 to 50%) and high risk (> 50%) of CBDS [49] (see Table 2). This proposed classification has clear clinical implications: patients with a low risk of CBDS should be operated on without further investigation; patients with moderate risk should be evaluated with a second-level examination, either preoperatively with endoscopic US (EUS) or magnetic resonance cholangiopancreatography (MRCP) or intraoperatively with laparoscopic US (LUS) or IOC, in order to select patients who need stone removal; finally, according to local expertise, laparoscopic transcystic CBD exploration is a valuable option. Patients with high risk of CBDS should undergo preoperative diagnostic and therapeutic ERCP. See Fig. 1 for the flowchart of management of ACC.

**Table 2:** Risk factors and classification of risk for CBDS (modified from Maple et al. 2010)

Very strong	Evidence of CBDS stone at the abdominal ultrasound
	Ascending cholangitis
Strong	Common bile duct diameter > 6 mm (with gallbladder in situ)
	Total serum bilirubin level > 1.8 mg/dl
Moderate	Abnormal liver biochemical test other than bilirubin
	Age older than 55 years
	Clinical gallstone pancreatitis
Risk class for choledocolithiasis	
High	Presence of any very strong
Low	No predictors present
Intermediate	All other patients

***What is the best tool to stratify the risk for CBDS in patients with ACC?***

**2.5 We suggest stratifying the risk of CBDS according to the proposed classification modified from the American Society of Gastrointestinal Endoscopy and the Society of American Gastrointestinal Endoscopic Surgeon Guidelines. #QoE: very low; SoR: weak#**

ASGE guidelines remains a valuable tool for the diagnosis and the management of CBDS in patients with ACC [49]. According to their classification, high-risk patients have a probability of having CBDS exceeding 50%, which in turn means that up to 49% of patients undergoing ERCP will not have evidence of CBDS and, given the potential complications of ERCP, this may not be considered acceptable. For this reason, we would recommend more cautious approach: only patients with evidence of CBDS at abdominal US should be considered at high risk of CBDS and should undergo diagnostic and therapeutic ERCP directly; patients with total serum bilirubin > 4 mg/dl or enlarged common bile duct diameter at US with concomitant bilirubin level 1.8 to 4 mg/dl should be considered as moderate risk and should undergo second level investigation such as endoscopic ultrasound (EUS) or magnetic resonance cholangiopancreatography (MRCP), laparoscopic ultrasound (LUS) or IOC, in order to avoid the complications related to ERCP. See Table 2 for the modified risk stratification.

***Which actions are warranted in patients with ACC and at moderate risk for CBDS?***

**2.6 We recommend that patients with moderate risk for CBDS undergo one of the following: preoperative magnetic resonance cholangiopancreatography (MRCP), preoperative endoscopic ultrasound (EUS), intraoperative cholangiography (IOC), or laparoscopic ultrasound (LUS), depending on local expertise and availability. #QoE: high; SoR: strong#**

Two preoperative imaging techniques are available for the detection of CBDS, namely MRCP and EUS. These diagnostic tests, according to the ASGE guidelines [49] should be reserved for patients with moderate risk for CBDS and have been shown to delay definitive ACC treatment [44]. On the other hand, these tests could exclude the presence of CBDS with high diagnostic accuracy, thereby avoiding further inappropriate invasive procedures, such as ERCP or IOC and therefore their complications. In fact, the implementation of these techniques resulted in a reduction of ERCP by 30 to 75% in non-selected patients [50, 51]. A Cochrane meta-analysis compared these two different techniques [52]: both had good diagnostic accuracy, showing summary sensitivities of 95% for EUS and 93% for MRCP and a summary specificity of 97% and 96%, respectively. As noted by some authors, considerations other than diagnostic efficacy, such as local availability, costs, expertise and delay of surgery, might play an important role in the decision making during the diagnostic work-up [53].

***Which actions are warranted in patients with ACC and at high risk for CBDS?***

**2.7 We recommend that patients with high-risk for CBDS undergo preoperative ERCP, IOC or LUS, depending on the local expertise and the availability of the technique. #QoE: high; SoR: strong#**

ERCP has both a diagnostic and a therapeutic role in the management of CBDS, but it is an invasive procedure with potential severe complications. The literature underscores the risks of diagnostic ERCP. Morbidity associated with diagnostic ERCP includes pancreatitis, cholangitis, bleeding, duodenal perforation and allergic reaction to contrast medium. Complications occur in 1 to 2% and increase to 10% when associated with sphincterotomy [54–57]. On the other hand, IOC significantly increases the length of surgery [58] and requires dedicated staff in the operating room, while this may not be available, especially in the acute setting with unplanned surgery. Positive findings on IOC often lead to intraoperative management of CBDS with additional operative time.

A recently published meta-analysis compared ERCP and IOC [58]. The summary sensitivity for ERCP was 0.83 (95% CI 0.72 to 0.90) and specificity was 0.99 (95% CI 0.94 to 1.00). For IOC, the summary sensitivity was 0.99 (95% CI 0.83 to 1.00) and specificity was 0.99 (95% CI 0.95 to 1.00). Sensitivities showed a weak statistical difference (*p* = 0.05); however, due to the low quality and the methodology of the included studies, the two diagnostic techniques should be considered equivalent. LUS is a useful method for intraoperative detection of CBDS [59]. A meta-analysis has shown that IOC and LUS have the same pooled sensitivity and similar pooled specificity for the detection of CBDS [60]. As in the case of IOC, intraoperative evidence of CBDS with LUS leads to intraoperative management of common bile duct with increased operative time.

***Which is the appropriate treatment of CBDS in patients with ACC?***

**2.8 We recommend removing CBDS, either preoperatively, intraoperatively, or postoperatively, according to the local expertise and the availability of several techniques. #QoE; high; SoR: strong#**

CBDS could be removed with several techniques and a variation of timing (see Fig. 1): preoperative ERCP with sphincterotomy, intraoperative ERCP with sphincterotomy, laparoscopic or open common bile duct exploration, post-operative ERCP with sphincterotomy. A systematic review assessed the differences between these techniques [61]. No differences in terms of morbidity, mortality and success rate were reported. Therefore, these techniques can be considered suitable options, depending on local expertise and availability. Another meta-analysis compared preoperative and intraoperative (rendez-vous technique) ERCP with sphincterotomy [62]. These two techniques were equal in terms of safety and efficacy; the intraoperative technique reduced the risk for post-ERCP pancreatitis, but required dedicated staff and prolonged the length of surgery.

## Section 3. Surgical treatment of ACC

The literature updated from the 2016 WSES Guidelines on ACC showed no remarkable publications to change the meaning of previous statements edited by the WSES in 2016 [7]; however, they have been reviewed to ensure the best available evidence.

***Which is the preferred first line of treatment for patients with ACC?***

***When should laparoscopic cholecystectomy be avoided in patients with ACC?***

**3.1 We recommend laparoscopic cholecystectomy as the first-line treatment for patients with ACC. #QoE: high; SoR: strong#**

Comment: A low complication rate and shortened hospital stay are the major advantages.

**3.2 We recommend avoiding laparoscopic cholecystectomy in case of septic shock or absolute anaesthesiology contraindications. #QoE: high; SoR: strong#**

Laparoscopic cholecystectomy is generally considered the standard technique for the removal of gallstones. Local inflammation, especially in gangrenous and emphysematous ACC, has been considered to increase the risk of bile duct injuries, blood loss, operative time, general morbidity and mortality rates in comparison with open surgery [63]. As technical difficulties usually decrease with experience and improvements in surgical technique and instrumentation, the hesitation to safely perform laparoscopic cholecystectomy in ACC has decreased over the years.

Despite several studies, ranging from case series to randomized prospective clinical trials, confirming the feasibility and safety of laparoscopic cholecystectomy in the treatment of patients with ACC [64–73], a recent survey on intra-abdominal infection, the CIAOW study [74], showed unexpected results. It was a worldwide survey of 68 medical institutions during a 6-month study period demonstrating that 48.7% of patients with ACC still underwent open surgery.

Nevertheless, evidence has clearly shown the safety of laparoscopic cholecystectomy in ACC. A recent systematic review, comparing open versus laparoscopic cholecystectomy, summarized the available evidence, underlining the limitations and providing a qualitative and quantitative analysis of the included studies. Of 651 studies, 10 were included after qualitative analysis (published between 1993 and 2012): four RCTs, two prospective non-randomized studies, and four retrospective trials, including 1374 patients (677 by laparoscopy vs. 697 by open surgery).

Laparoscopic cholecystectomy in ACC was associated with a lower complication rate and with a shorter hospital stay. There were no differences for the same-admission cholecystectomy in terms of morbidity, operative time and intraoperative blood loss and bile leakage; however, the laparoscopic approach showed a decrease in mortality rate, postoperative hospital stay, wound infection and pneumonia. Moreover, the operative time progressively became shorter in laparoscopy when data were analysed its instances between 1998 and 2007 [75].

A reaffirmation of the safety of laparoscopic cholecystectomy for ACC was shown in another systematic review comparing early laparoscopic cholecystectomy (ELC) and delayed laparoscopic cholecystectomy (DLC), including seven discordant meta-analyses and systematic reviews published from 2004 to 2015. The conclusions were that no differences in mortality, bile duct injury, bile leakage, overall complications and conversion to open surgery were seen. However, ELC had a significant reduction in wound infection, hospitalisation, duration of surgery and quality of life [76].

TG18 widened the indications for laparoscopic cholecystectomy when compared with TG13, as they supported same-admission laparoscopic cholecystectomy for patients with all three severity grades of ACC [77, 78]. This is in line with the recommendations of the 2016 WSES Guidelines [7].

In summary, the review of the relevant recent literature confirmed strong support for the recommendation that laparoscopic cholecystectomy should be attempted in cases of ACC. Critical patient conditions, such as septic shock or anaesthesiology contraindication, are reasons to avoid laparoscopic cholecystectomy.

***Is laparoscopic cholecystectomy safe and feasible for patients with ACC who have liver cirrhosis, are older than 80 years or are pregnant?***

**3.3 We suggest performing laparoscopic cholecystectomy for ACC patients with Child’s A and B cirrhosis, patients with advanced age (including more than 80 years old) and patients who are pregnant. #QoE: low; SoR: weak#**

### Patients with liver cirrhosis

In cases of liver cirrhosis, surgical dissection could be difficult and there is a higher risk of bleeding and other serious complications. Unfortunately, the available evidence on both open and laparoscopic cholecystectomy for ACC in patients with liver cirrhosis is limited. Therefore, we mainly accept evidence that comes from elective procedures performed for biliary colic or chronic cholecystitis. According to a meta-analysis published by de Goede et al., elective laparoscopic cholecystectomy in patients with child A or B cirrhosis was associated with significantly fewer postoperative complications, a shorter duration of hospitalisation and a shorter time to resume a normal diet, when compared to the open technique [79]. Lucidi et al. recommended laparoscopic cholecystectomy as the first-choice approach in cirrhotic patients. However, recommendation for laparoscopic cholecystectomy in patients with child C cirrhosis is unclear [80]. Nevertheless, other studies showed that laparoscopic cholecystectomy in these cirrhotic patients was associated with a significantly prolonged duration of surgery and an increase in operative blood loss, conversion rate, length of hospital stay and overall morbidity and mortality when compared with non-cirrhotic patients [81]. In cirrhotic patients, the morbidity associated with laparoscopic cholecystectomy is directly related to the Child-Pugh score [82, 83].

In patients with advanced cirrhosis and severe portal hypertension, specific technical difficulties may be encountered due to the presence of a portal cavernoma, the difficulty in dissecting the Calot’s triangle and the gallbladder hilum, the presence of adhesions and neovascularization or difficulty in controlling bleeding from the liver bed. Subtotal cholecystectomy is a valid option to avoid some of these difficulties [84, 85].

In conclusion, the laparoscopic approach should be the first choice for cholecystectomy in child A and B patients. The approach to patients with child C or uncompensated cirrhosis remains a matter of debate. As a first recommendation, cholecystectomy should be avoided in these patients, unless clearly indicated, such as in ACC not responding to conservative management [80].

### Patients over 80 years old

The true clinical relevance of age is difficult to assess and the impact of old age on the clinical outcomes in cases of surgical abdominal pathology is largely unknown.

In 2017, the WSES and the Italian Society for Geriatric Surgery developed a CC and a consequent set of guidelines on this topic. Only retrospective studies have focused their interest on elderly patients with ACC. In general, no RCTs are available, population sizes of the studies were small and distributed over a long period of time. It should be noted that the prevalence of elderly people with ACC could increase in the future, due to the improvement in life expectancy and the consideration that the risk of biliary stones increases with age. Some of the recommendations were derived from evidence describing the general population, which includes the elderly. The level of evidence for surgery, timing and risk assessment ranged from 2 to 3, and the grade of recommendation ranged from B to C according the 2011 Oxford classification “(https://www.cebm.net/wp-content/uploads/2014/06/CEBM-Levels-of-Evidence-2.1.pdf.)”. With these limitations, the conclusion supported laparoscopic cholecystectomy for ACC in elderly patients, after considering the intrinsic surgical risk, life expectancy and the rate of relapse in cases of conservative management; and frailty scores, in the absence of a single universally accepted score, were evaluated as adjunctive tools to better characterize elderly patients in the clinical situation [9].

More recently, Wiggins et al. have published a retrospective study based on an administrative national database of all consecutive patients aged over 80, who were admitted for ACC in England between 1997 and 2012. It included a very large number of patients (47,500). On index admission, non-operative treatment was carried out for 89.7% of the patients. Then, 7.5% had a cholecystectomy, and the remaining 2.8% had a cholecystostomy. The three groups were slightly different in mean age (83, 85 and 85 years, respectively) and the Charlson Comorbidity Index was below 2 in 87.5%, 83.1% and 83.2%, respectively. When surgery was compared to non-operative management (NOM) and to cholecystectomy, the mortality rate showed a trend favouring surgical management. The 30-day mortality rates were 11.6% for surgery, 9.9% for NOM (*p* < 0.001) and 13.4% for cholecystectomy (*p* < 0.001); the 90-day mortality rates were 15.6% for surgery, 16.1% for NOM (*p* > 0.05) and 22.5% for cholecystectomy (*p* < 0.001); the 1-year mortality rates were 20.8% for surgery, 27.1% for NOM and 37% for cholecystostomy (*p* < 0.001). It should be noted that this study showed a readmission rate of more than 50% after conservative management, which probably contributed to the increased mortality rate at 90 days and 1 year in this group. Interestingly, the proportion of cholecystectomies performed laparoscopically increased from 27 to 59% between 2006 and 2012. Moreover, multivariate analysis showed that, among the surgical group at the index admission, laparoscopy played an independent protective role, with an 84% relative risk reduction in 30-day mortality (OR 0.16, 95% CI 0.10–0.25) when compared to open cholecystectomy. In the discussion, the authors pointed out that the results could have some relationship with the fact that they came from a nation in which early cholecystectomy in ACC patients, regardless of age, is applied only in 15.7% of cases, as compared to 52.7% in the USA [86].

With a decreased cut-off at 70 years old for the definition of elderly patients, the safety of ELC in ACC has also been supported by Loozen et al. in a systematic review and meta-analysis published in 2017. The cumulative morbidity and mortality were 24% and 3%, respectively, and there was a higher rate of complications for non-elderly patients. The protective role of laparoscopy is therefore confirmed; however, the authors highlight that there are limitations to their findings, in that there was an absence of prospective studies included in the review [11].

In conclusion, despite the low quality of evidence, the studies detailed here universally favour ELC for elderly patients, even for patients older than 80 years of age. Due to the generally small functional reserve in the elderly, care should be taken to ensure that a prompt therapeutic decision is taken and that a high level of expertise is provided, both intraoperatively and during the postoperative management.

### Patients who are pregnant

The literature evidence for pregnant patients is limited. The incidence of ACC during pregnancy varies among reports, ranging from one case per 1600 pregnancies to one case per 10,000 pregnancies. However, ACC during pregnancy is the second reason for non-obstetrical abdominal emergency surgery after appendicitis [87, 88].

The diagnostic criteria and tools are the same used for the general population [89], but it is of note that leucocytosis during pregnancy could be misleading, and that the Murphy sign could be difficult to evaluate in the late part of the third trimester.

The best option for the management of ACC should be chosen considering a balance among the following factors: the risk of complications from ACC, limitations on medication availability depending on the trimester, the risk of relapse, the risk of other specific conditions which may occur during pregnancy and the time until delivery or maturation of the foetus.

In general, in the absence of contraindications, surgery is suggested as first-line therapy in order to avoid complications and potential drug toxicity for the foetus. Retrospective studies stated that recurrent ACC or pancreatitis can occur in 10% of patients, while miscarriage can occur in 10–20% of patients [90]. NOM is an alternative option, but it must be highlighted that there is a risk of higher incidence of spontaneous abortion, threatened abortion, and premature birth when compared to patients who underwent cholecystectomy [91].

One systematic review and meta-analysis focused on the comparison of open cholecystectomy with laparoscopic cholecystectomy during pregnancy [92]. The authors selected 11 studies, all of which were retrospective: two from national databases, one from a state database and the others were retrospective from single- or multiple- institutions. The analysis included 10,632 patients (1219 open; 9413 laparoscopic). The outcomes were as follows:

- Outcomes for the mother: death occurred in 1 open versus 0 laparoscopic cases; complications (including caesarean section, dilatation and curettage, hysterectomy, maternal dehydration and pre-eclampsia) were 3.5% in laparoscopic cases vs. 8.2% in open cases, with an odds ratio (OR) of 0.42 (95% CI 0.33–0.53, *p* < 0.001).
- Outcomes for the foetus: 1 death out of 161 patients (0.6%) in laparoscopic cases versus 4 deaths from 93 patients (4.3%) in open cases, with an OR of 0.39 (95% CI 0.07–2.19, *p* = 0.29); the complications (including foetal loss, foetal distress, threatened preterm delivery and preterm birth) were 346 out of 8807 laparoscopic cases (3.9%) vs. 139 out of 1161 open cases (12.0%), with an OR of 0.42 (95%; 0.28–0.63, *p* < 0.001).
- Surgical complications (including bile duct injury, bile duct leaking, solid organ or hollow viscus injury, pulmonary and wound infections, and hernias): 901 out of 9413 laparoscopic cases (9.6%) versus 211 out of 1219 open cases (17.3%), with an OR of 0.45 (95% CI 0.25–0.82, *p* = 0.01).
- Preterm delivery (< 37-week gestation): 11 out of 127 laparoscopic cases (8.7%) versus 5 out of 78 open cases (6.4%), with an OR of 1.35 (95% CI 0.41–5.14; *p* = 0.59).
- The Apgar score at 5 minutes was the same.

In 2018, another Japanese nationwide retrospective cohort study reported similar results [93].

With the limitations of the quality of the studies, laparoscopy should be suggested for the treatment of symptomatic gallstones including ACC. The vast majority of studies suggests the second trimester until the initial part of the third trimester as the best time to perform laparoscopic cholecystectomy, as there is a higher risk of miscarriage and toxic effect of anaesthesia in the first trimester, while concerns are related to the size of the uterus in the third trimester [92–94].

A systematic review and set of guidelines from the British Society for Gynaecological Endoscopy, endorsed by the Royal College of Obstetricians and Gynaecologists, have been published in 2019 and they summarize the discussion, confirming the benefits of ELC over non-operative treatment, especially in the second trimester [95].

***Which surgical strategies should be adopted in case of difficult anatomic identification of structures during cholecystectomy for ACC?***

**3.4 We recommend laparoscopic or open subtotal cholecystectomy in situations in which anatomic identification is difficult and in which the risk of iatrogenic injuries is high. #QoE: moderate; SoR: strong#**

Reasons for a ‘difficult gallbladder’ vary, and can be related to obesity, adhesions, acute or chronic inflammation, distended gallbladder and liver cirrhosis. Due to the diversity of reasons and the variability of approaches among surgeons, a review conducted in 2011 showed no consensus on the ideal way to deal with a difficult gallbladder. The options include subtotal cholecystectomy, fundus first cholecystectomy, perioperative cholangiogram, open conversion or a combination of these options [96].

In this section, we focus on subtotal cholecystectomy, which is an option when the critical view of safety [97] cannot be obtained. In 2015, a systematic review and meta-analysis by Elshaer et al. reported that subtotal cholecystectomy was performed using laparoscopic (72.9%) open (19.0%) and laparoscopic converted to open (8.0%) techniques. In this study including over 1200 patients, the most common indications were severe cholecystitis (72.1%), followed by gallstones in liver cirrhosis and portal hypertension (18.2%) and empyema or a perforated gallbladder (6.1%). They concluded that subtotal cholecystectomy might be helpful during the surgical management of difficult cholecystectomy; also considering that it achieves morbidity rates comparable to those reported for total cholecystectomy in straightforward cases. The quality of evidence is limited, due to the absence of prospective randomized studies, which are not expected to be easily performed in the future [85].

Support for subtotal cholecystectomy has also been reported from other studies. In a retrospective study on severely difficult gallbladders [98], 105 patients who underwent laparoscopic cholecystectomy were matched with 46 patients who underwent subtotal laparoscopic cholecystectomy. The authors observed no bile duct injury in the subtotal cholecystectomy group, but four instances in the complete cholecystectomy group. Bile leakage was greater in the subtotal group due to difficulty in the cicatrisation on the remaining gallbladder stump; however, bile leakage was managed easily by abdominal drainage or in combination with endoscopic biliary prosthesis placement.

A recent nation-based database study evaluating 290,855 cases between 2003 and 2014 showed an increased use of subtotal cholecystectomy from 0.1 to 0.52% for open subtotal cholecystectomy and from 0.12 to 0.28% for laparoscopic cholecystectomy. The conversion rate from laparoscopic to open total cholecystectomy decreased from 10.5 to 7.6%. Interestingly, the teaching hospitals significantly increased the rate of subtotal cholecystectomy [99]. Furthermore, it should be highlighted that there are different techniques to achieve subtotal cholecystectomy: this aspect could add some difficulty in analysing data from different studies [100].

The quality of the available evidence ranges from low to moderate. However, the concordance of all the evidence, the large application of the technique globally, with the important clinical impact on patient safety, and the current absence of opportunities to achieve a better level of evidence strongly supports the recommendation for subtotal cholecystectomy in cases of difficult gallbladder.

***When should conversion from laparoscopic to open cholecystectomy be considered in patients with ACC?***

**3.5 We recommend conversion from laparoscopic to open cholecystectomy in case of severe local inflammation, adhesions, bleeding from the Calot’s triangle or suspected bile duct injury. #QoE: moderate; SoR: strong#**

This recommendation should be supported by studies in which the patients with difficult gallbladder have been randomized to conversion or to different laparoscopic procedures. However, this type of study is unlikely to be performed.

The present update of the WSES Guidelines on ACC clarifies that, in 2016, the reasons for conversion were used as a proxy in the absence of high-quality studies; we maintained the same approach for the current version of the guidelines [7, 101, 102].

When expertise in difficult instances of laparoscopic cholecystectomy is ensured, the conversion is not a failure and it represents a valid option to be considered. The quality of the evidence is moderate. However, the absence of opportunities to achieve a higher quality of evidence, along with the broadly used conversion to open surgery and the clinical impact on patients’ safety, suggests a strong recommendation for conversion to open surgery after laparoscopy has been attempted at the best institutional level available. Nevertheless, according to Gupta et al., surgeons should adopt a philosophy of safe laparoscopic cholecystectomy. Understanding the mechanisms related to specific complications may help elaborating strategies to avoid or reduce those complications; in this context, the surgeon should define, in their own personal armamentarium, the indications for a bailout techniques among the available options [103, 104].

## Section 4. Timing of cholecystectomy in people with ACC

***When is the optimal timing for laparoscopic cholecystectomy in patients with ACC?***

**4.1 In the presence of adequate surgical expertise, we recommend ELC be performed as soon as possible, within 7 days from hospital admission and within 10 days from the onset of symptoms. #QoE: moderate; SoR: strong#.**

Comment: ELC so defined is preferable to intermediate laparoscopic cholecystectomy (ILC, performed between 7 days of hospital admission and 6 weeks) and DLC (performed between 6 weeks and 3 months).

**4.2 We suggest DLC to be performed beyond 6 weeks from the first clinical presentation, in case ELC cannot be performed (within 7 days of hospital admission and within 10 days of onset of symptoms). #QoE: very low; SoR: weak#.**

Surgery is currently the recommended treatment in people with acute cholecystitis. Conservative management with fluids, analgesia and antibiotics is an option for people with mildly symptomatic acute cholecystitis (i.e. people without peritonitis or those who have worsening clinical condition). In a RCT with long-term follow-up of 14 years, about 30% of patients treated conservatively developed recurrent gallstone-related complications and 60% of patients had undergone cholecystectomy subsequently [105, 106]. The study included only 33 patients and had high risk of bias [105, 106]. Therefore, until new high-quality evidence becomes available, laparoscopic cholecystectomy is considered the recommended treatment for patients who are fit to undergo surgery.

In patients with moderate or severely symptomatic cholecystitis or in those with mildly symptomatic acute cholecystitis who prefer surgery, laparoscopic cholecystectomy is preferred over open cholecystectomy [75]. The timing of laparoscopic cholecystectomy in these patients is controversial. A Cochrane review published in 2013 concluded that ELC for acute cholecystitis seems safe and may shorten the total hospital stay [107].

An update of the literature searches was performed for the purpose of this guideline. Sixteen trials were identified in the update (including the trials originally included in the systematic review) [108–123]. The number of participants with acute cholecystitis was not reported in one of the trials [121]. In the remaining 15 trials, 1240 participants were included in 14 trials comparing ELC versus DLC [108–115, 117–120, 122, 123] and 618 participants were included in one trial comparing ELC versus intermediate laparoscopic cholecystectomy (ILC) [116]. The country; recruitment period; number of participants; the duration of symptoms; the timing of ELC, DLC and ILC; and the surgical experience are reported in Table 3. Overall, it appears that ELC was performed within 10 days of onset of symptoms in most trials.

**Table 3:** Timing of cholecystectomy in people with ACC

Study name	Timing of surgery in early group	Number of participants in early group	Timing of surgery in intermediate or delayed group	Number of participants in intermediate or delayed group	Risk of bias^a^
Davila 1999 (1)	< 4 days after diagnosis	27	2 months after discharge	36	Unclear
Gul 2013 (2)	< 72 h after hospital admission	30	6 to 12 weeks after initial conservative treatment	30	High
Gutt 2013 (3)	< 24 h after hospital admission	304	7 to 45 days after hospital admission^2^	314	Low
Johansson 2003 (4)	< 7 days of diagnosis	74	6 to 8 weeks after discharge	71	Low
Kolla 2004 (5)	< 24 h after randomisation	20	6 to 12 weeks after the acute episode subsides	20	Low
Lai 1998 (6)	< 24 h after randomisation	53	6 to 8 weeks after the acute episode subsides	51	Low
Lo 1998 (7)	< 72 h after admission	45	8 to 12 weeks after discharge	41	High
Macafee 2009 (8)	< 72 h after recruitment	Not stated	3 months after discharge	Not stated	High
Mustafa 2016 (9)	< 48 to 72 h of diagnosis	105	6 to 12 weeks after initial attack	105	High
Ozkardes 2014 (10)	< 24 h of admission	30	6 to 8 weeks after initial treatment	30	High
Rajcok 2016 (11)	< 72 h after occurrence of symptoms	32	6 to 8 weeks after acute cholecystitis	32	High
Roulin 2016 (12)	During day as soon as possible	42	6 weeks after initial diagnosis	44	High
Saber 2014 (13)	< 72 h of duration of symptoms	60	6 to 8 weeks from onset of symptoms	60	High
Verma 2013 (14)	< 72 h of admission	30	6 to 8 weeks from onset of symptoms	30	High
Yadav 2009 (15)	As soon as possible	25	6 to 8 weeks after discharge	25	High
Zahur 2014 (16)	< 24 to 48 h after hospital admission	47	6 to 8 weeks after initial conservative treatment	41	High

Main reasons for unclear or high risk of bias

High risk of bias: at least one of random sequence generation, allocation concealment, missing outcome bias or selective outcome reporting bias was classified as high risk of bias

Unclear risk of bias: at least one of random sequence generation, allocation concealment, missing outcome bias or selective outcome reporting bias was classified as unclear risk of bias without any of the domains being classified as high risk of bias

^a^All studies were at high risk of bias due to lack of blinding. The risk of bias classification stated here is for the remaining domains

^b^This was the only study in which intermediate laparoscopic cholecystectomy was performed; delayed laparoscopic cholecystectomy was performed in the remaining studies

There were no significant differences in mortality or conversion to open cholecystectomy between the three groups. The proportion of patients with serious adverse events was significantly higher in ILC compared to ELC in the only trial included in the comparison between ILC and DLC [116]. The number of serious adverse events was significantly less with ELC compared to DLC in the only trial comparing ELC with DLC that reported this information [111]. The total length of hospital stay (including all the admissions for treatment) was about 4 days shorter with ELC compared to DLC [109–112, 115, 117–120, 123], and about 5 days shorter with ELC compared to ILC [116]. The return to work was about 9 days sooner following ELC compared to DLC [109, 120].

Overall, it appears that ELC performed within 7 days of hospital admission and within 10 days of onset of symptoms is superior to either ILC performed between 7 days of hospital admission and 6 weeks or DLC performed between 6 weeks and 3 months of the initial hospital admission for acute cholecystitis. Since blinding cannot be achieved in these comparisons and the outcomes were subjective, all the trials were deemed to be at high risk of bias. However, trials with low risk of bias are difficult to conduct in this comparison. Since the evidence was consistent across trials and outcomes, it appears highly likely that ELC is superior to either ILC or DLC. Therefore, despite the moderate quality evidence (which is mainly because of the lack of blinding in the trials), the recommendation for ELC is strong. However, it should be noted that the study authors described that ELC was more complex; therefore, it should be attempted only by experienced surgeons. Referral to centres with high surgical expertise should be considered if adequate surgical expertise is not available. If ELC cannot be performed, DLC appears to be better than ILC. Although, there is no evidence of difference between DLC and ILC, the ACDC trial comparing ELC versus ILC showed that a significant proportion of patients undergoing ILC developed serious adverse events [116]. Therefore, DLC may be preferable when ELC is not possible, although a proportion of patients with planned DLC approach may need unplanned earlier surgery (see Fig. 1) [107]. There are no trials comparing ILC and DLC and it is unlikely that they will performed, given the results of the ACDC trial [116]. Therefore, there is significant uncertainty whether DLC is better than ILC when ELC is not possible and the recommendation to perform DLC when ELC is not possible is weak.

## Section 5. Risk prediction in ACC

***How can the prognosis and surgical risk be assessed for patients with ACC?***

**5.1 We cannot suggest the use of any prognostic model in patients with ACC.**

**#QoE: very low; SoR: weak#**

Comment: There is currently significant uncertainty in the ability of prognostic factors and risk prediction models in predicting outcomes in patients with ACC.

Cholecystectomy is currently the recommended treatment for patients with acute cholecystitis. Laparoscopic cholecystectomy is preferred over open cholecystectomy in patients with acute cholecystitis, but it is a major surgical procedure. While it is considered relatively safe, it is associated with a mortality rate between 0.1 and 1% [124–126], a risk of bile duct injury in approximately 0.2% to 1.5% of cases [125, 127] and a risk of major complications (such as myocardial infarction, heart failure, acute stroke, renal failure, pulmonary embolism, lung failure or postoperative shock) in between 6 and 9% of cases [124].

Observation is an alternative option for patients with mildly symptomatic ACC (i.e. in patients without peritonitis or in those who have worsening symptoms). After a long-term follow-up of 14 years, about 30% of patients with mildly symptomatic acute cholecystitis who did not undergo cholecystectomy developed recurrent gallstone-related complications, compared with 3% of patients who underwent cholecystectomy. These differences were not significant for recurrent disease or overall complications [128]. However, 60% of patients had undergone surgery, and the study was small and carried a high risk of bias; therefore, there is lot of uncertainty as to whether it is better to perform surgery or observation in patients with mildly symptomatic acute cholecystitis.

Identification of patients at high risk of complications and mortality can help in optimising them prior to surgery or in deciding whether referral to high-volume centres and specialized centres, which may decrease the complications [129, 130], is appropriate. Informed decisions about whether to opt for surgery or observation can also be made if information on the risks is available.

We performed a systematic review of studies reporting on the ability of prognostic factors or risk prediction models to predict important patient-related outcomes, such as mortality, complications and conversion to open surgery in patients with ACC [131]. In this systematic review and meta-analysis, we included 12 studies and 6827 patients in one or more analysis. Only a few factors (TG13, age, male gender, previous abdominal surgery, diabetes, hypertension and C-reactive protein) were reported in a format similar enough to allow comparisons between studies. The remaining factors were studied in single studies or using different thresholds. Therefore, there is no information on their reproducibility, and the results may be unreliable.

Among the prognostic factors reported in at least two studies, TG13 grade 3 had an increased risk of all-cause mortality compared to grade 1. The risk increased from a median risk of 1.3% to 6.5% (95% CI 3.7–11.2). However, most studies included only people who underwent surgery, not all of whom were patients with ACC. There have been no RCTs of surgery versus observation in people with severe ACC. Laparoscopic cholecystectomy performed by experienced surgeons had lower major complication rates than percutaneous cholecystostomy with no planned cholecystectomy [11]. Therefore, it appears that, despite the increased risk of mortality in TG13 grade 3 compared to TG13 grade 1, surgery seems to be the preferred option when possible. However, referral to high volume centres and specialized centres may decrease the complications [129, 130] and resulting mortality, and should be considered in people with TG13 grade 3 acute cholecystitis.

Being male was associated with an increased risk of complications (from 10 to 15%; 95% CI 10.5–20.9) and an increased risk of conversion to open cholecystectomy (from 16 to 48.5%; 95% CI 27.5–70.0). The reasons for the difference in the complications and conversion between males and females are not clear, but may be due to a combination of increased skeletal muscle mass [132] (particularly in the trunk [133]) and increased visceral abdominal fat in males [132, 134, 135], which could make laparoscopic surgery more difficult, and a common delay in males seeking medical help due to a misguided perception of masculinity [136, 137], which could mean that the males had more severe disease than females at the time of presentation to the hospital. Previous upper abdominal surgery is a risk factor for conversion to open cholecystectomy. This is to be expected because of the intra-abdominal adhesions related to previous abdominal surgery [138]. An increased age had a minor increase in the conversion to open cholecystectomy, but the increase is cumulative, as elderly patients may have a clinically significant increase in conversion to open cholecystectomy compared with young people. Various confounding factors such as comorbidities and increased cumulative risk of upper abdominal surgery may contribute to the increased risk of conversion to open cholecystectomy.

However, it should be noted that the systematic review included only preoperative factors, and most of the studies included only patients undergoing cholecystectomy for ACC. Therefore, the findings of the review are applicable only for preoperative risk prediction in patients undergoing cholecystectomy for ACC.

It should also be noted that most of the studies were retrospective, in which blinding of predictor or outcome measurement were not reported, and most of the studies were small.

Overall, there is significant uncertainty in the ability of prognostic factors and risk prediction models in predicting outcomes in patients with ACC. TG13 grade 3 may be associated with greater mortality than grade 1 severity of acute cholecystitis. Despite the increased risk of mortality in TG13 grade 3 compared to TG13 grade 1, surgery seems to be the preferred option when possible. The TG18 adopted the TG13 severity grading criteria in predicting outcomes in patients with ACC [14].

High-quality studies are necessary to provide better information on the prognostic factors of patients with acute cholecystitis and to improve shared decision making.

## Section 6. Alternative treatment for patients with ACC who are not suitable for surgery: observation and techniques for gallbladder drainage

***When should Non-Operative Management be considered for patients with ACC?***

**6.1 We suggest considering NOM, i.e. best medical therapy with antibiotics and observation, for patients refusing surgery or those who are not suitable for surgery. #QoE: low; SoR: weak#**

**6.2 We suggest considering alternative treatment options for patients who fail NOM and who still refuse surgery or patients who are not suitable for surgery. #QoE: low; SoR: weak#**

Schimdt et al. [105] published an RCT comparing observation and surgery in cases of ACC, with a long median follow-up time of 14 years. In their analysis, about 30% of patients with mildly symptomatic acute cholecystitis who did not undergo cholecystectomy developed recurrent gallstone-related complications, as compared with 3% of patients who underwent cholecystectomy, but these differences were not significant for recurrent disease or complications. Overall, 60% of patients had undergone surgery, while 40% avoided surgery. There are significant limitations in the study, as recognized by the authors: firstly, a relevant percentage of eligible patients (41%) were excluded from randomization; secondly, the reasons for the exclusion were not stated in the paper; thirdly, the definitions of dropout and failure within the observation group were not clear. Brazzelli et al. [139] published a systematic review of two RCTs comparing observation with surgery in patients with symptomatic gallstone disease (in the first study) and patients with ACC (in the second study). From a total of 201 patients, the results confirmed the high rate of gallstone-related complications within the observation group (RR 6.63, 95% CI 1.57–28.51, *p* = 0.01).The authors, although noting the substantial lack of good quality evidence, reported that a policy of surgery for all patients with ACC, when compared to a policy of observation followed by surgery for symptomatic patients, represents a costly but more effective choice. In conclusion, relevant uncertainty exists regarding the best management between surgery or observation in cases of ACC, especially in uncomplicated disease; observation and best medical therapy are likely to be safe, but this latter approach is characterised by a high incidence of recurrent disease.

Alternative treatment options may be considered for patients who fail NOM for ACC, also considering the individual patient’s characteristics and the clinical situation.

***Which is the first-choice treatment for ACC in high risk patients?***

**6.3 Immediate laparoscopic cholecystectomy is superior to percutaneous transhepatic gallbladder drainage (PTGBD) in high risk patients with ACC. We recommend laparoscopic cholecystectomy as the first-choice treatment in this group of patients. #QoE: high; SoR: strong#**

TG13 on ACC [140] considered gallbladder drainage as mandatory for patients with severe grade ACC (according to the Tokyo classification [10] of acute cholecystitis) and also suggested its use in the moderate grade if conservative treatment fail. The revised TG18, based on recent studies, proposed that severe-grade cholecystitis, under certain strict criteria, may be treated with laparoscopic cholecystectomy [10, 140]. A systematic review published in 2016 comparing percutaneous transhepatic gallbladder drainage (PTGBD) and cholecystectomy in critically ill patients reported no benefit for the use of PTGBD over cholecystectomy [141]. Six studies were analysed with a total of 337,500 patients. Mortality rate, length of hospital stay and number of readmissions for gallstone-related diseases were all significantly higher in the PTGBD group than in the cholecystectomy group. It should be noted that all included studies had a retrospective design, which makes the results prone to bias. Recently, the first randomized trial on this subject was published (the CHOCOLATE trial). The results showed that laparoscopic cholecystectomy is superior to PTGBD [11] also in high-risk patients with ACC. PTGBD was compared with ELC in critically ill patients (APACHE score 7–14) with ACC, in terms of efficacy and safety. Patients who underwent ELC had significantly fewer major complications, which were mainly recurrent biliary events. Five percent of patients who underwent ELC had complications, compared with 53% of patients who underwent PTGBD. Mortality was low and remained the same in both groups.

Early laparoscopic cholecystectomy also led to significantly less utilisation of health care resources. The trial concluded that immediate cholecystectomy in high-risk patients is safe and should be the standard of care.

***What is the role of gallbladder drainage in patients with ACC who are not suitable for surgery?***

**6.4 We recommend performing gallbladder drainage in patients with ACC who are not suitable for surgery, as it converts a septic patient with ACC into a non-septic patient. #QoE: moderate; SoR: strong#**

Patients who are not suitable for surgery, but who are septic due to gallbladder empyema, are effectively treated by PTGBD, as shown in the CHOCOLATE trial [11]. Gallbladder drainage decompresses the infected bile or pus in the gallbladder, removing the infected collection without removing the gallbladder. The removal of the infected material can result in reduced inflammation and in improvement of the clinical conditions. Several case series, both retrospective and observational, exist on cholecystostomy. A systematic review of the literature included 53 studies with 1918 patients outlining a high success rate of the procedure (85.6%) with a low procedure-related mortality rate (0.36%); however, the 30-day mortality rate was high at 15.4% [142]. A major limitation of the study was the inclusion of patients with both acute acalculous cholecystitis and ACC. A review of additional 27observational studies on cholecystostomy [143] showed significant heterogeneity in terms of inclusion criteria, results and conclusions reached by different authors. Considering these limitations, the reported in-hospital mortality and morbidity rates for cholecystostomy range from 4 to 50 % and from 8.2 to 62%, respectively.

Gallbladder drainage may be an option in patients who failed conservative management after a variable time of 24 to 48 h and who present with strict contraindications for surgery. A prospective study by Barak et al. [144] reported that age over 70 years, diabetes, tachycardia and a distended gallbladder at admission are predictors for failure of NOM at 24-h follow-up, while a WBC of more than 15,000 cell/mm^3^, fever and age above 70 years were predictors for failure of NOM at 48-h follow-up.

No specific antibiotic regimen is to be prescribed alongside PTGBD and no evidence exists supporting the need for a specific antibiotic regimen. For the antimicrobial therapy, please refer to the dedicated section.

***Should delayed cholecystectomy be offered to patients with ACC after the reduction of perioperative risk?***

**6.5 Delayed laparoscopic cholecystectomy is suggested after reduction of perioperative risks to decrease readmission for ACC relapse or gallstone-related disease. #QoE: very low; SoR: weak#**

De Mestral et al. published a large retrospective epidemiologic analysis in 2013, showing that 40% of patients underwent a DLC after PTGD; the 1-year readmission rate for patients who did not undergo DLC after PTGD was 49% with an in-hospital mortality rate of 1% [145]. No randomized trial currently exists comparing DLC to observation alone.

***Can endoscopic gallbladder drainage be considered an alternative to PTGBD in patients with ACC who are not suitable for surgery?***

**6.6 In patients with ACC who are not suitable for surgery, endoscopic transpapillary gallbladder drainage (ETGBD) or ultrasound-guided transmural gallbladder drainage (EUS-GBD) should be considered safe and effective alternatives to PTGBD, if performed in high-volume centers by skilled endoscopists. #QoE: high; SoR: strong#**

ACC is a frequent event in urgent surgical settings, and the gold standard for its treatment is laparoscopic cholecystectomy [140, 146, 147]. However, some patients are unfit for surgery, and for them non-surgical drainage represents a suitable option, either as a bridge to subsequent surgery, once their clinical conditions improve, or as a definitive treatment for those who remain poor candidates for surgery. Non-surgical approaches include PTGBD and endoscopic procedures. Among these, alternatives are endoscopic transpapillary gallbladder drainage (ETGBD), with placement of a transpapillary naso-gallbladder drainage tube (ENGBD) or double-pigtails tent (EGBS), or transmural ultrasonography-guided gallbladder drainage (EUS-GBD) [148].

In a systematic review about options for endoscopic gallbladder drainage, Itoi et al. [149] reported a pooled technical success rate of ENGBD of 80.9% (95% CI 74.7–86.2) and a pooled clinical response rate of 75.3% (95% CI 68.6–81.2%). For EGBS, the results were 96% (95% CI 91.1–98.7) and 88% (95% CI 81.2–93.2), respectively. At that time, only small case-series were available for EUS-GBD, with reported technical and clinical success rates of 100% (95% CI 75.3–100).

Five years later, Itoi et al. [150], in a RCT of 73 consecutive patients with ACC, obtained overall technical success rates with ENGBD and EGBS of 91.9% and 86.1%, respectively, whereas the clinical success rates by intention-to-treat analysis were 86.5% and 77.8%, respectively. The authors argued that the lower clinical success rate may be ascribed to inadequate gallbladder drainage when large stones or pus were present, and to the use of small-diameter catheters or stents.

EUS-GBD has been compared, in terms of technical feasibility and efficacy, to PTGBD [151] inpatients with acute, high-risk or advanced stage cholecystitis who did not respond to initial medical treatment and could not undergo ELC. EUS-GBD and PTGBD showed similar technical (97% vs. 97%, *p* = 0.001) and clinical (100% vs. 96%, *p* = 0.0001) success rates, and similar rates of complications (7% vs. 1%, *p* = 0.492), indicating that EUS-GBD is a safe alternative to PTGBD in patients who are unsuitable for surgery.

Irani and co-workers [152] reached similar conclusions after a retrospective multicenter study, in which the technical success rates of EUS-GBD and PTGBD were 98% and 100% (*p* = 0.88), respectively. Moreover, the EUS-GBD group had a shorter in-hospital length of stay and fewer repeat interventions (*p* < 0.05).

Khan et al. [153], in a meta-analysis, evaluated the technical success rates and post-procedure adverse events of ETGBD compared with PTGBD. They found that the pooled OR for technical success of ETGBD versus PTGBD was 0.51 (95% CI 0.09–2.88; *I*^2^ = 23%) and for post-procedures adverse events was 0.33 (95% CI 0.14–0.80; *I*^2^ = 16%) in favour of ETGBD. The weighted pooled rates (WPRs) for EUS-GBD were as follows: technical success 93% (95% CI 87–96; *I*^2^ = 0%), clinical success 97% (95% CI 93–99; *I*^2^ = 0%), post-procedure adverse events 13% (95% CI 8–19; *I*^2^ = 0%) and recurrent cholecystitis 4% (95% CI 2–9; *I*^2^ = 0%).

In a prospective study on long term-follow up after EUS-GBD [154], the recurrence of cholecystitis was observed in 7.7% of cases, suggesting that this endoscopic procedure is a safe alternative in the treatment of acute cholecystitis in high-risk patients.

EUS-GBD has also been proven to be a feasible technique for the conversion of percutaneous cholecystostomy [155]. The advantages of EUS-GBD over PTGBD include an internalization of bile, obviating the risk of recurrent cholecystitis following percutaneous catheter removal and the risk of bleeding, and being associated with less post-procedural pain [155, 156].

A recent RCT by Teoh et al. [157] identified patients with ACC at very high risk for surgery as patients older than 80 years, with an ASA grade ≥ 3 or an age-adjusted Charlson Comorbidity score > 5 and/or a Karnofsky score < 50. The authors randomized them to receive either EUS-GBD with LAMS or PTGBD within 4 to 6 h from diagnosis. Although 30-day mortality was equivalent between the two study groups, the results were in favour of EUS-GBD, which was associated with less adverse events at 30-day (12.8% vs. 47.5%, *p* = 0.001) and at 1-year follow-up (25.6% vs. 77.5%, *p* < 0.001), with a reduced number of re-interventions at 30 days (2.6% vs. 30%, *p* = 0.001) and with a reduced number of episodes of recurrent cholecystitis (2.6% vs. 20%, *p* = 0.029).

**6.7 If endoscopic transpapillary gallbladder drainage is performed, both endoscopic nasogatric endoscopic gallbladder drainage (ENGBD) and endoscopic gallbladder stenting (EGBS) should be considered suitable options, based on patient characteristics and on the endoscopist’s decision. #QoE: high; SoR: strong#**

Although ENGBD has certain advantages for patients in whom stent insertion is impossible or when there is stent dysfunction, it has two major drawbacks: the potential for inadvertent catheter dislodgement or patient removal, and discomfort. On the other hand, an indwelling stent may be suitable when there is a concern in patients with altered mental status or dementia [6]. A meta-analysis conducted in TG18 [140] found no statistically significant difference in technical success (OR 1.18; 95% CI 0.36–3.89), clinical success (OR 1.82; 95% CI 0.40–8.26), or adverse events rate (OR 1.49; CI 95% 0.29–3.81). Consequently, the advantages and disadvantages of each drainage method are considered approximately equal.

***What is the role of endoscopic transmural ultrasound-guided gallbladder drainage (EUS-GBD) in patients with ACC who are not suitable for surgery?***

**6.8 EUS-GBD with lumen-apposing self-expandable metal stents (LAMSs) should be preferred to ETGBD, if performed by skilled endoscopists. #QoE: moderate; SoR: strong#**

In a recent meta-analysis, Khan et al. [153] found that the proportional difference of WPRs for technical success and clinical success between EUS-GBD versus ETGBD were 10% and 4%, respectively. This difference is explained by the fact that the transpapillary procedures may be technically challenging, due to the anatomy of the cystic duct or stone impaction. On the other hand, if the distance between the gallbladder and the enteric lumen is less than 1 cm, EUS-GBD appears to be safe and feasible [154–156]. This technique results in a permanent fistula formation between the gallbladder and the hollow viscus, facilitating anatomic bile drainage [158].

A variety of plastic stents (straight, single, double-pigtail) and self-expandable metal stents (SEMSs) have been used during EUS-GBD with similar treatment outcomes. However, plastic and SEMSs are tubular stents not specifically designed for EUS-GBD procedures; therefore, bile leakage, stent occlusion and migration are potential adverse events [148, 159]. In order to overcome these limitations, modified stents with flared ends and LAMSs have been introduced [159, 160].

LAMSs are fully covered self-expandable metal stents with bilateral flanges specifically designed for EUS-guided, trans-enteric drainage of a pseudocyst or non-adherent fluid collections [148]. The theoretical advantage of LAMSs over other stents is the ability to approximate the gallbladder wall to the enteric lumen, ‘sealing off’ any potential bile leaks and preventing migration, thus providing a robust lumen anchorage [160]. Furthermore, the large diameter of LAMSs (10 mm and 15 mm) may allow access to the gallbladder with a slim endoscope with the purpose of removing stones, or taking biopsies [148].

In a retrospective review of multi-center prospectively collected data, Irani et al. [160] achieved a technical success rate of 93% and a clinical success rate of 100% using LAMSs to decompress the gallbladder in patients who had ACC and who were poor surgical candidates. Dollhopf et al. [161], with a new LAMSs device, obtained technical and clinical success rates of 98.7% and 95.9%, respectively, with 10.7% of cases having adverse events.

**6.9 If a EUG-GBD is performed using metal stents, we recommend their removal within 4 weeks, in order to avoid food impaction with subsequent high risk of recurrence of ACC. #QoE: low; SoR: weak#**

The long-term deployment of metal stents in EUS-GBD could cause adverse events, including food impaction, which, by impairing bile flow, may induce recurrence of cholecystitis. There are several evidences [159, 162] that a well formed fistula might develop between the gallbladder and the gastrointestinal tract within four weeks using a conventional biliary SEMS as well as a LAMS.

Some authors [162] have argued that, in order to minimise the risk of recurrent cholecystitis or biliary leakage, LAMSs could be left in place for a longer period, without stent-related complications [13]. Although a more significant tissue reaction can be expected after a long stented well-time, it seems probable that stent location, whether gastric or duodenal, might also influence the degree of tissue overgrowth. The retroperitoneal location of the duodenum results in a more stable tract to the gallbladder than the stomach, in which peristaltic movements might result in a more pronounced tissue reaction, impairing the removal of the stent once the inflammatory process has subsided.

## Section 7. Antibiotic treatment of ACC

***Which is the optimal antibiotic treatment for patients with uncomplicated ACC?***

**7.1 In uncomplicated ACC, we recommend against the routine use of postoperative antibiotics when the focus of infection is controlled by cholecystectomy. #QoE: high; SoR: strong#**

An open-label non-inferiority prospective controlled trial by Regimbau et al. [163] randomized 414 patients who underwent cholecystectomy for uncomplicated ACC to either no antibiotics after surgery or continuation of the preoperative antibiotic regimen for 5 days. An imputed intention-to-treat analysis showed no difference in the incidence of postoperative infection rates: 17% (35 out of 207) in the no-treatment group compared with 15% (31 out of 207) in the antibiotic group (absolute difference 1.93%; 95% CI − 8.98–5.12).

No studies were found on this topic since the publication of the 2016 WSES Guidelines on ACC.

***Which is the optimal antibiotic treatment for patients with complicated ACC?***

**7.2 In complicated ACC, we recommend prescribing the antimicrobial regimen based on the presumed pathogens involved and the risk factors for major resistance patterns. #QoE: high; SoR: strong#**

Empiric antibiotic treatment should be commenced according to the most frequently isolated microorganisms, taking into consideration the local trends of antibiotic resistance and the availability of drugs. In biliary infections, Gram-negative aerobes, such as *Escherichia coli* and *Klebsiella pneumonia*, and anaerobes, especially *Bacteroides fragilis* are the most commonly isolated bacteria [74, 164]. The potential pathogenicity of Enterococci in biliary sepsis remains unclear and specific coverage against these microorganisms is not routinely suggested for community-acquired biliary infections [165]. In case of immunosuppression, i.e. transplant patients, infection lead by *Enterococcus* spp. should be presumed and pre-emptively treated [166]. The main issue related to antibiotic resistance in biliary tract infections remains the production of extended spectrum beta-lactamase by Enterobacteriaceae; this is frequently found in community acquired infections in patients with previous exposure to antibiotics [74, 164].

Healthcare-related infections are commonly caused by resistant bacterial strains, requiring complex antibiotic regimens; the use of adequate broad-spectrum empiric therapy appears to be a crucial factor to reduce postoperative complications and deaths, especially in critically ill patients [166]. The efficacy of antibiotics in the treatment of biliary infections may be associated with their biliary concentration, although few clinical or experimental data exists supporting the use of antibiotics with biliary penetration for these patients. Nevertheless, in patients with obstructed bile duct, the biliary penetration of antibiotics may be poor and the actual bile concentrations are reached only in a small percentage of patients [167]. Biliary penetration of different antibiotics (indicated as the ratio of bile-to-serum concentrations) are listed in Table 4 [168].

**Table 4:**
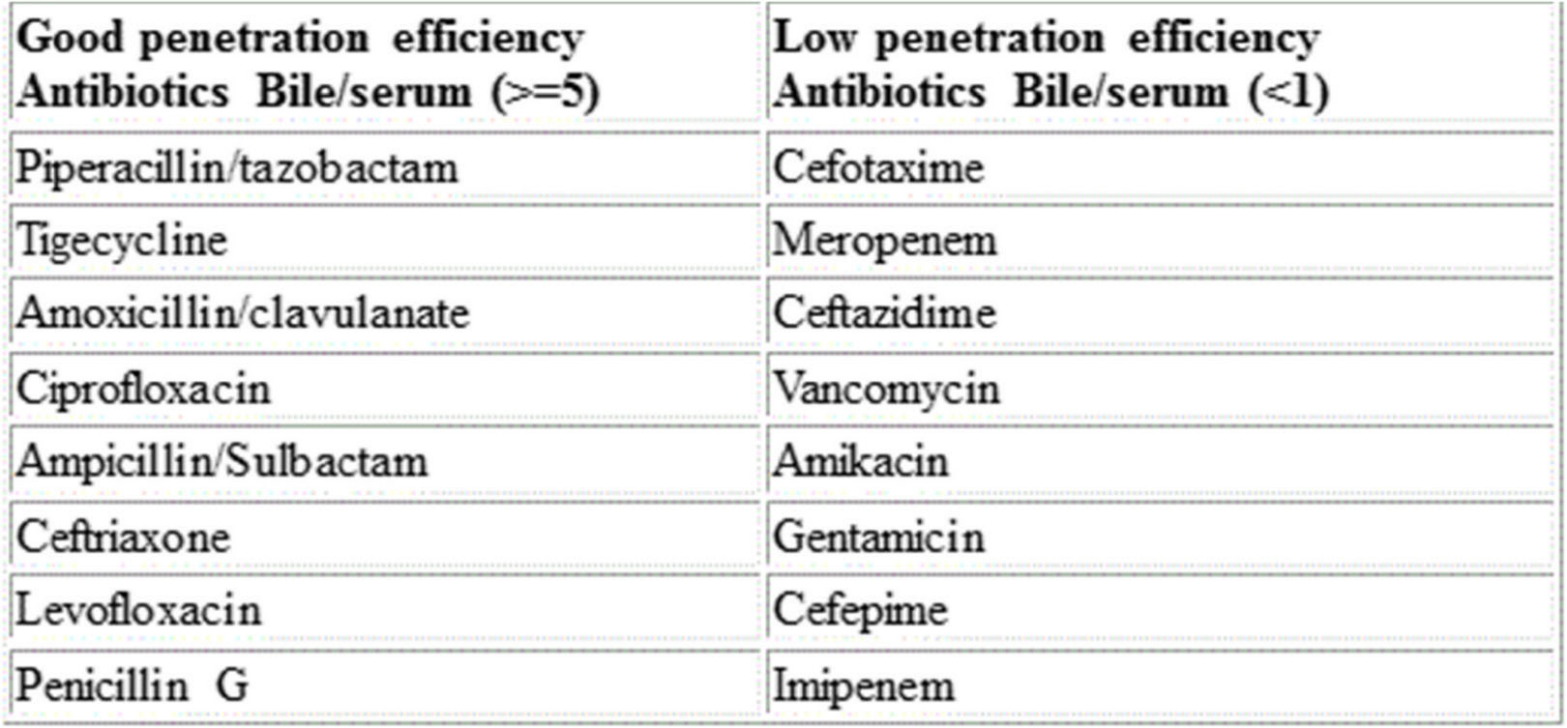
Antimicrobial regimens suggested for ACC

In the management of critically ill patients with ACC, the choice of the antimicrobial regimen may be challenging. In patients with sepsis of abdominal origin, the early administration of a correct empirical antimicrobial therapy has a significant impact on outcome [169]. Richè et al. prospectively studied a cohort of 180 consecutive patients with secondary generalized peritonitis and found that there was a significantly higher mortality rate in patients with septic shock than in those without septic shock (35% and 8%, respectively, OR 4.11; 95% CI 1.78–9.48, *p* = 0.0003) [170]. Furthermore, in patients with septic shock, the biliary origin of peritonitis was a risk factor for mortality at multivariate analysis (OR 3.5; 95% CI 1.09–11.70, *p* = 0.03). International guidelines for the management of severe sepsis and septic shock (the Surviving Sepsis Campaign) [171] recommend the administration of broad-spectrum intravenous antibiotics with good penetration into the presumed site of infection within the first hour. A recent CC (Sepsis-3) [172] proposed a new evidence-based definition of sepsis and septic shock, underscoring the importance of recognizing the septic focus and the infecting organism. In cases of biliary sepsis, drug pharmacokinetics may be significantly altered in patients with organ dysfunction and septic shock; therefore, the selection of antibiotics should be reassessed daily and be based on both the pathophysiological status of the patient and the pharmacokinetic properties of the specific drug [173]. No significant additional evidence was found since the publication of the 2016 WSES Guidelines on ACC (see Table 4 for recommended antibiotic regimens).

***What is the role of microbiological cultures and sensitivities in patients with ACC?***

**7.3 In patients with complicated ACC and patients at high risk for antimicrobial resistance, we recommend adapting the targeted antibiotic regimen to the results of microbiological analysis, ensuring adequate antimicrobial coverage. #QoE: moderate; SoR: weak#**

Identifying the causative organism(s) is an essential step in the management of ACC, especially in patients at high risk for antimicrobial resistance, such as immunocompromised patients and those with healthcare-associated infections. The rate of positive bile culture (from either gallbladder culture or bile samples from the common bile duct) ranges from 29 to 54% in cases of ACC [174–179].

No additional studies have been found on this topic since the publication of the 2016 WSES Guidelines on ACC.

## Conclusions, knowledge gaps and research recommendations

The WSES 2020 on ACC, based on the updated evidence, reinforces the pivotal role of ELC in the management of ACC, even in high-risk patients. The new developed algorithm, in our opinion, emphasizes the importance of two categories of patients: the high-risk patients and those who are not suitable for surgery.

The CHOCOLATE Study [11] defined high-risk patients as those with an APACHE score 7–14; this high-quality study improved our understanding of the management of this complex cohort of patients. Its results are in favour of surgery, when compared with biliary drainage in high-risk patients with ACC. However, there is not a single and universally accepted definition of this high-risk patients group; therefore, accepting the suggestion coming from Loozen et al., it appears reasonable to recommend the development of local clinical pathways after deciding which of the available scores fits local needs and expertise.

In addition to the defined high-risk patients, the WSES proposes the category of patients who are not suitable for surgery. We suggest to include in this group all patients with ACC who are not fit for surgery, according to the specific surgeon’s judgement, and patients who are not amenable for surgical treatment, due to the presence of clinical conditions which are not classifiable with clinical or physiologic scores (Appendix 2). For this cohort of patients, surgery may be unsafe or impossible and gallbladder drainage may be the best suitable option in case of uncontrolled sepsis and/or failure of NOM.

Moreover, areas for important future research were identified. These include (1) high-quality studies on prognostic factors of ACC patients so as to improve shared decision making, (2) defining the best management option when ELC is not possible due to lack of surgical expertise or due to the duration of symptoms. This should include focus groups involving patients and clinicians, and using observational studies, and (3) defining the best option in the management of difficult operative scenarios. This needs a common language among researchers in order to obtain higher quality studies (in terms of classification of difficulties: e.g. adhesions with hollow viscus, difficulties in grasping the gallbladder, difficulties in view of safety, gangrene of the cystic duct, etc.).

Finally, the WSES strongly advocates the adoption of a policy for safe laparoscopic cholecystectomy and encourages the development of local pathways, based on the available evidence.

## Supplementary information

**Additional file 1.** Appendix 1 and 2

## Data Availability

All data generated or analysed during this study are included in this published article [and its supplementary information files].

## Abbreviations

ACC: Acute calculous cholecystitis
GRADE: Grading of Recommendations Assessment, Development and Evaluation
TG: Tokyo Guidelines
CC: Consensus Conference
CBDS: Common bile duct stone
RCT: Randomized controlled trial
LR: Likelihood ratio
US: Ultrasound
pSWE: Point shear-wave elastography
AUC: Area under the curve
SMI: Superb microvascular imaging
HIDA: Hepatobiliary iminodiacetic acid
CT: Computed tomography
MRI: Magnetic resonance imaging
ERCP: Endoscopic retrograde colangio-pancreatography
LFTs: Liver function tests
GGT: Gamma-glutamyl transpeptidase
ALT: Alanine aminotransferase
IOC: Intra-operative cholangiography
PPV: Positive predictive value
NPV: Negative predictive value
ALP: Alkaline phosphatase
EUS: Endoscopic ultrasound
MCRP: Magnetic resonance colangio-pancreatography
LUS: Laparoscopic ultrasound
ELC: Early laparoscopic cholecystectomy
OR: Odds ratio
95% CI: 95% confidence interval
ILC: Intermediate laparoscopic cholecystectomy
DLC: Delayed laparoscopic cholecystectomy
NOM: Non operative management
PTGBD: Percutaneous transhepatic gallbladder drainage
ETGBD: Endoscopic transpapillary gallbladder drainage
EUS-GBD: Ultrasound-guided transmural gallbladder drainage
ENGBD: Endoscopic transpapillary nasogastric gallbladder drainage
EGBS: Endoscopic gallbladderstent
LAMS: Lumen-apposing self-expandable metal stent
SEMS: Self-expandable metal stents
