# Supplementary material for: 2020 World Society of Emergency Surgery updated guidelines for the diagnosis and treatment of acute calculus cholecystitis
Source: World J Emerg Surg. 2020 Nov 5;15:61. doi: 10.1186/s13017-020-00336-x (PMC7643471; doi:10.1186/s13017-020-00336-x)
Supplement: Supplementary file 1 — Additional file 1. Appendix 1 and 2 [file 13017_2020_336_MOESM1_ESM.docx]

## Appendix 1. Summary of Statements

The appendix 1 presents the summary of statements of the 2020 World Society of Emergency Surgery Updated Guidelines for the Diagnosis and Treatment of Acute Calculus Cholecystitis: scientific evidence supporting the GRADE are briefly reported. Authors recommend, for a detailed analysis, the use of the body text of the present guidelines.

| ***Statement from the guidelines*** | ***Quality of Evidence*** | ***Recommendation and reasons*** |
| --- | --- | --- |
| **Which is the most reliable approach for the diagnosis of ACC?**   - 1. As no feature has sufficient diagnostic power to establish or exclude the diagnosis of ACC, it is recommended not to rely on a single clinical or laboratory finding.     1.2 For the diagnosis of ACC, we suggest using a combination of detailed history, complete clinical examination, laboratory tests and imaging investigations. However, the best combination is not known. | High  Very low | Strong   - High quality evidence: one systematic review and meta-analysis of 17 studies and one prospective study of >400 patients, detailing diagnostic values of the clinical signs and laboratory tests; strong study designs and consistent evidence supporting the statement. (Trowbridge RL et al. 2003, Eskelinen M et al. 2004) - Relative effects of the tests are not in the same direction, as each test has its own drawbacks and merits   Weak   - Very low-quality evidence: no studies regarding the effect of the combination of the stated signs and tests, with poor study design (Janikow C et al. 2017, Naidu K et al. 2016, Joseph B et al. 2018) - There is variability in information (one of the retrospective analysis does not fully support the statement with a suggested accuracy for diagnosis of 60.4%, well below the TG13 report -(Naidu K et al. 2016) - No evidence on costs and utilisation of resources, which would be important for this statement. |
| **Which initial imaging technique should be used in case of a suspected diagnosis of ACC?**  1.3 We recommend the use of abdominal ultrasound (US) as the preferred initial imaging technique, in view of its cost-effectiveness, wide availability, reduced invasiveness and good accuracy for gallstones disease.  *Comment: Abdominal US is a reliable investigation method; however, it may be of limited utility to rule in or rule out the diagnosis of ACC according to the adopted US criteria.* | High | Strong   - High quality evidence: one systematic review and meta-analysis (Kiewec. et al 2012) with relevant heterogeneity, but strong study design; eight observational studies with weak study design were assessed. Six out of eight had a sample size <300, however they all support the use of US as the preferential imaging technique. ( - Cost and utilisation of resources favour ultrasound. - High patient importance |
| **Which is the role of other imaging techniques (e.g. HIDA scan, Abdominal CT scan and Magnetic Resonance Imaging) in the diagnosis of ACC?**  1.4 We suggest the use of further imaging for the diagnosis of ACC in selected patients, depending on local expertise and availability. Hepatobiliary iminodiacetic acid (HIDA) scan has the highest sensitivity and specificity for the diagnosis of ACC as compared to other imaging modalities. Diagnostic accuracy of computed tomography (CT) is poor. Magnetic resonance imaging is as accurate as abdominal US.  *Comment: in clinical practice HIDA scan is limited due to the required resources and time* | Moderate | Strong   - Moderate quality evidence: one retrospective analytic study with 412 patients (Changphaisarnkul P et al. 2015), two prospective studies with 101 patients and 126 patients (Fagenzholz et al. 2015, Kim SW et al. 2015), one retrospective study with 81 patients (Carter SS et al. 2017) and one retrospective review of 1217 patients (Kaoutzanis C et al. 2015). Results on CT-scan and MRI are heterogenous; however, the studies are consistent on HIDA - Variability in information: conflicting results regarding the accuracy of ultrasound and CT-scan with opposite information in two studies - Relative effects not in the same direction; each technique has specific advantages and drawback - Cost and utilisation of resources are not considered |
| **Are elevated LFTs or bilirubin sufficient for the diagnosis of CBDS in patients with ACC?**  2.1 We recommend against the use of elevated of liver biochemical enzymes or bilirubin as the only method to identify CBDS in patients with ACC. We therefore recommend performing further diagnostic tests. | Moderate | Strong   - Moderate quality evidence: prospective and retrospective cohort/observational studies or case series; weak study designs (Peng WK et al. 2005, Barkun AN et al. 1994, Onken JE et al. 1996, Song SH et al. 2014, Chang C et al. 2009, Padda MS et al. 2009). However, consistent evidence with a large sample size >400 - No variability in information - Costs and utilisation of resources not considered |
| **Which imaging features are predictive of CBDS in patients with ACC?**  2.2 We suggest considering the visualization of a stone in the common bile duct at transabdominal ultrasound as a predictor of CBDS in patients with ACC.  2.3 An increased diameter of common bile duct, an indirect sign of stone presence, is not sufficient to identify ACC patients with CBDS and we therefore recommend performing further diagnostic tests. | Very low  High | Weak   - Very low-quality evidence: one retrospective review of 248 patients, reporting that ultrasound is not sufficient to identify patients at a significant risk for CBDS (Boys JA et al. 2014). Weak study design and small sample size - Insufficient number of studies to support the statement   Strong   - High quality evidence: one meta-analysis of five retrospective/prospective studies including 523 patients (Gurusamy K et al. 2015) and one retrospective review of 248 patients (Boys JA et al. 2014); both studies consistently support the statement - No variability in information as studies suggest low sensitivity; hence, the need for further tests - Costs and utilisation of resources are not considered |
| **Which tests should be performed to assess the risk of CBDS in patients with ACC?**  2.4 In order to assess the risk for CBDS, we suggest performing liver function tests (LFTs), including ALT, AST, bilirubin, ALP, gamma glutamyl transferase (GGT) and abdominal US in all patients with ACC. | Low | Weak   - Low quality evidence: retrospective studies (Soltan HM et al. 2001), reviews (Sarli L et al. 2000), guidelines (Maple JT et al. 2010) and prospective cohorts (Menezes N et al. 2000; Sun X et al. 2003); overall weak study designs and small sample sizes. Furthermore, indirect evidence as all the studies are not specific for ACC - Costs and utilisation of resources not considered |
| **Which is the best tool to stratify the risk for CBDS in patients with ACC?**  2.5 We suggest stratifying the risk of CBDS according to the proposed classification modified from the American Society of Gastrointestinal Endoscopy and the Society of American Gastrointestinal Endoscopic Surgeon Guidelines. | Very low | Weak   - Very low-quality evidence: evidence from guidelines based on expert consensus (Maple JT at al. 2010) - Weak recommendation as no high quality of evidence studies test the clinical effects of the modified classification (no external validation) - Unclear absolute/relative effects - Costs and utilisation of resources not considered |
| **Which actions are warranted in patients with ACC and at moderate for CBDS?**  2.6 We recommend that patients with moderate risk for CBDS undergo one of the following: preoperative magnetic resonance cholangiopancreatography (MRCP), preoperative endoscopic ultrasound (EUS), intraoperative cholangiography (IOC), or laparoscopic ultrasound (LUS), depending on local expertise and availability. | High | Strong   - High quality evidence: Cochrane meta-analysis of 18 studies with 2366 patients (Giljaca V et al. 2015) and ASGE guidelines (Maple JT et al. 2010). The studies consistently report high diagnostic accuracy - Values were presented in favour of the statement: studies suggest high diagnostic accuracy, e.g. 96% for EUS, 93% MRCP; no variability in the information given by studies - Strong absolute effect - Costs and utilisation of resources not applicable as statement suggests dependent on availability |
| **Which actions are warranted in patients with ACC and at high risk for CBDS?**  2.7 We recommend that patients with high risk for CBDS undergo preoperative ERCP, intraoperative cholangiography or LUS, depending on the local expertise and the availability of the technique. | High | Strong   - High quality evidence: two meta-analyses (Aziz O et al. 2014, Dasari BV et al. 2013) consistently suggests all four procedures do not have significant difference; similar sensitivity and specificity with good overlap of CI, with high diagnostic accuracy - No variability or absence of information - Costs and utilisation of resources not applicable |
| **Which is the appropriate treatment of CBDS in patients with ACC?**  2.8 We recommend removing CBDS, either preoperatively, intraoperatively or postoperatively, according to the local expertise and the availability of several technique. | High | Strong   - High quality evidence: one systematic review of 16 RCTs with 1758 patients (Dasari BV et al. 2013) and one meta-analysis of five RCTs with 531 patients (Wang B et al. 2013). The two meta-analyses consistently support the statement. RCTs may be imprecise due to the sample size but all five gave consistent results with high baseline risk. - No variability in information - Costs and utilisation of resources not applicable |
| ***Which is the preferred first line of treatment for patients with ACC?***  ***When should laparoscopic cholecystectomy be avoided in patients with ACC?***  3.1 We recommend laparoscopic cholecystectomy as a first-line treatment for patients with ACC.  3.2 We recommend avoiding laparoscopic cholecystectomy in case of septic shock or absolute anaesthesiology contraindications. | High | Strong   - High quality evidence: - one systematic review and meta-analysis of 4 RCTs, 2 prospective nonrandomized studies, and 4 retrospective trials with a total of 1374 patients.   (Coccolini et al. 2015)   - cumulative analysis of 7 discordant meta-analysis and systematic reviews from 2004 to 2015. (Song et al. 2016) - two international guidelines (Okamoto, et al 2018; Ansaloni et al. 2016) - The differences between laparoscopy and open surgery is well analysed for different items. Laparoscopy is supported in Coccolini study by the 4 RCT included, by the large number of patients and using proxy when needed. - The concern in performing laparoscopy in case of shock is less scientifically consistent however it arise from all the studies considered and from consensus of experts in both international guidelines. - The study detailed the difference between laparoscopy and open surgery according to several items. The study was focused on drawbacks and merits of native studies; however, the presence of 4RCT, the large number of included patients, the accordance to other proxy favouring laparoscopy, |
| ***Is laparoscopic cholecystectomy safe and feasible for patients with ACC who have liver cirrhosis, are older than 80 years or are pregnant?***  3.3 We suggest laparoscopic cholecystectomy for ACC patients with Child A and B cirrhosis, patients with advanced age (including more than 80 years old) and patients who are pregnant. | Low | Cirrhosis:  Weak   - Low quality evidence: - one meta-analysis of 4 RCT   (de Goede B et al.2013)   - one RCT and observational studies (Lucidi et al. 2009) - one meta-analysis tincluding 1 RCT (Puggioni et al 2003) - Despite meta-analysis and, RCT the quality of evidence is poor due to the limitation and incompleteness of data, few number of patients.   Extrapolation of information from study designed for immunoresponse.   - Age>80-year-old:   Weak   - Low quality evidence coming from retrospective studies with small group of patients, summarized in an international guideline. Despite the low quality of studies, the published reports support early laparoscopic surgery. (Pisano et al. 2019) - Low quality evidence from a retrospective and administrative database. The quality of the study is reinforced by a very large population study size; however one more limit is that the surgical arm is largely smaller than the NOM ones reflecting the discrepancy of England respect to other Nations in the rate of early surgery for ACC. (Wiggins et al. 2018) - Low quality evidence from a systematic review and meta-analysis of retrospective studies with moderate risk of bias. With these limitations also this cumulative analysis also supports early laparoscopic surgery. (Loozen et al. 2017) - of early surgery for ACC. (Wiggins et al. 2018) - Low quality evidence from a systematic review and meta-analysis of retrospective studies with moderate risk of bias. With these limitations also this cumulative analysis also supports early laparoscopic surgery. (Loozen et al. 2017)   Pregnancy:  Weak   - Low quality of evidence from a systematic review and meta-analysis of 11 retrospective studies, some of these based on administrative database. Adjunctive An additional limit is that the studies are exclusively from USA. However, the power is reinforced by the large populations study size of 10,632 patients; the results support early laparoscopic approach to symptomatic gallbladder stone disease, including ACC, especially in the second trimesterthe results suggest, especially in the second trimester, early laparoscopic approach to symptomatic gallbladder stone disease, including ACC. (Sedaghat et al. Surg Endosc 2017 31:673–679) - Low quality of evidence from a retrospective national (Japan) analysis. The cholecystectomy group included ACC patient, assessed with a separate subgroup analysis from database; the group of cholecystectomy, included ACC related, was a subgroup of the analysis. The results suggest early laparoscopic approach.   (Shigemi et al. Journal of Minimally Invasive Gynecology 2019; 26 (3): 501-506)   - Low quality of evidence from a national (UK) guideline that underlined the low quality of studies; with these limitations the evaluations of the available evidence support the early laparoscopic cholecystectomy during the second trimester of pregnancy instead of stand of NOM. (Ball et al. Facts Views Vis Obgyn 2019; 11 (1): 5-25) |
| ***Which surgical strategies should be adopted in case of difficult anatomic identification of structures during cholecystectomy for ACC?***  3.4 We recommend laparoscopic or open subtotal cholecystectomy in situations in which anatomic identification is difficult and in which the risk of iatrogenic injuries is high | Moderate | Strong   - Moderate quality of evidence from a systematic review and meta-analysis: the studies were all retrospective; however the global quality of the results must be classified moderate due to the well performed selections of included studies, the large population study (more than 1200) looking at this particular technique, and the well conducted analysis for several items; in favour to moderate is the consistency of the results for subtotal cholecystectomy. (Elsher et al. 2015). - Low quality of evidence due to the retrospective nature of the study; however, the simple size (105 patients), respect to the topic, is adequate for a single institution. The results are largely in favour to subtotal cholecystectomy. (Purzner et al. 2019). - Low quality of evidence due to the retrospective nature of the study; the study doesn’t focus on safety and complications of subtotal cholecystectomy but on the national (USA) diffusion of the technique and the increasing usage of it: this could suggest the validity of the laparoscopic subtotal cholecystectomy in case of difficult gallbladder. (Sabour et al. 2019) |
| ***When should conversion from laparoscopic to open cholecystectomy be considered in patients with ACC?***  3.5 We recommend conversion from laparoscopic to open cholecystectomy in case of severe local inflammation, adhesions, bleeding from the Calot’s triangle or suspected bile duct injury | Moderate | Strong   - Low quality of evidence due to the use of proxy from retrospective studies; absence of well-constructed study focused on the comparison between laparoscopic techniques vs. conversion to open in case of difficult gallbladder. Of note this topic is reasonably not eligible for RCT. |
| **Which is the optimal timing for laparoscopic cholecystectomy in patients with ACC?**  4.1 In the presence of adequate surgical expertise, we suggest ELC be performed as soon as possible within 7 days of hospital admission and within 10 days of onset of symptoms.  4.2 We suggest DLC beyond 6 weeks, in stand of ILC, in case of impossibility to perform ELC within 7 days of hospital admission and within 10 days of onset of symptoms. | Moderate  Very low | Strong   - Moderate quality evidence: Chocrhane metaanalysis (Gurusamy 2013). Updated systematic review and metaanalysis by the same author of the Chocrane review (Gurusamy 2013) for the purpose of the present guideline (unpublished data): 16 studies included (please see Table 3 for details) - Despite most studies have a very small sample size, weak study design and high risk of bias without blinding, they present consistent information supporting the statement - High patient importance due to the better quality of life of patients using ELC (less hospital stay and quicker return to work) - Cost and resources considered and favourable to ELC   Weak   - Very low-quality evidence: extrapolation of information from one study in a systematic review (Macafee et al. 2009) - no direct evidence comparing DLC and ILC. - The extrapolation of information leads to indirectness of evidence - Unclear relative effects as DLC itself poses with earlier unplanned surgery |
| **How can be preoperatively assessed the risk for patients with ACC?**  5.1 We cannot suggest the use of any prognostic model in patients with ACC. | Very low | Weak   - Very low-quality evidence: under review of systematic review of 12 studies (Tufo 2020; accepted for publication in Journal of Laparoendoscopic & Advanced Surgical Techniques.) - Large number of patients: 6827. Most of the studies are retrospective studies with a small sample size. These studies had a weak study design and did not mention blinding of predictors or outcome measure, making them susceptible to bias. - Other studies mentioned did not address the question directly but there was an extrapolation of information, as a study was focused on differences in whole body skeletal mass by gender. (Abe T et al. 2003) - Variability of importance as studies focus on different criteria that leads to different outcomes |
| **When should Non-Operative Management (NOM) be considered for patients with ACC?**  6.1 We suggest considering NOM, i.e. best medical therapy with antibiotics and observation, for patients refusing surgery or those who are not suitable for surgery.  6.2 We suggest considering alternative treatment options for patients who fail NOM and who still refuse surgery or patient who are not suitable for surgery. | Low | Weak   - Low quality evidence: one RCT (Scmidt M et al. 2011) and one systematic review (Brazzelli M et al. 2015) of two RCTs with a small sample size (<300). Weak study designs (major flaws: unclear randomisation and unclear exclusion criteria), with limited patient number. - It exists variability on the outcomes from different studies. Mortality rate reported in the systematic review did not show significant differences between the two groups. - Costs and resources are considered; however it is unclear what specific aspects are taken into account |
| **Which is the first-choice treatment for ACC in high-risk patients?**  6.3 Immediate laparoscopic cholecystectomy is superior to percutaneous gallbladder drainage (PTGBD) in high risk patients with ACC; we recommend laparoscopic cholecystectomy as a first-choice treatment in this group of patients. | High | Strong   - High quality evidence: one systematic review (Ambe PC et al. 2016) of six studies -no RCTs included, with 337500 patients suggests PD is not-superior and even inferior to cholecystectomy. Studies are homogenous with I^2^ >70% and no overlap of CI. One RCT ((Loozen et al. 2018) with 142 patients shows laparoscopic cholecystectomy to be superior to PD - Strong absolute effect, as systematic review shows OR >4 for mortality associated with PC. Significant shorter hospital stay and less readmission rates for patients managed with cholecystectomy - Variability of information in terms of mortality as systematic review suggests superiority of cholecystectomy but in the RCT there are no significant differences between the two options. - Costs and resources are considered in the RCT, suggesting early laparoscopic cholecystectomy reduced costs and resources |
| **Which is the role of gallbladder drainage in** **patients with ACC who are not suitable for surgery?**  6.4 We recommend performing gallbladder drainage in septic patients with ACC who are not suitable for surgery, as it converts a septic patient with ACC into a non-septic patient. | Moderate | Strong   - Moderate quality evidence: one RCT of 142 patients, one systematic review of 53 studies with 1918 patients (Winbladh et al. 2009; most of the included studies were retrospective studies/case series) and one systematic review of 27 observational studies (Campanile FC et al. 2014) - Relative effects not in the same direction as RCT suggests percutaneous gallbladder drainage have higher risk of complications/readmissions. High success rate, low procedure-related mortality but high 30-day mortality (Loozen et al. 2018) - Costs and resources are not considered, but marginal interest |
| **Should delayed cholecystectomy be offered to patients with ACC after the reduction of perioperative risk?**  6.5 Delayed laparoscopic cholecystectomy is suggested after reduction of perioperative risks to decrease the risk of acute readmission for ACC relapse or gallstone-related disease. | Very low | Weak   - Very low-quality evidence: one retrospective epidemiological analysis (De Mestral C et al. 2013) with 14220 patients, with weak study design - No comments on variability of information is formulated. |
| **Can endoscopic gallbladder drainage be considered an alternative to PTGBD in patients with ACC who are not suitable for surgery?**  6.6 In patients with ACC who are not suitable for surgery, endoscopic transpapillary gallbladder drainage (ETGBD) or ultrasound-guided transmural gallbladder drainage (EUS-GBD) should be considered safe and effective alternatives to PTGBD, if performed in high-volume institutes by skilled endoscopists.    6.7 If ETGBD is performed, both endoscopic nasogatric endoscopic gallbladder drainage (ENGBD) and endoscopic gallbladder stenting (EGBS) should be considered suitable options, based on patient characteristics and on the endoscopist’s decision | High  High | Strong   - High quality evidence: two systematic reviews (Ho J et al. 2018, Anderloni et al. 2016), two RCTs (Itoi T et al. 2015, Jang JW et al. 2012), one retrospective studies of 90 patients (Irani S et al. 2017) and one case series of 7 patients (Law R et al. 2016). - Relevant heterogeneity in one of the systematic reviews (I^2^ < 40%), but minimal overlap of CI. Publication bias not described in the reviews. Overall, consistent information supporting the statement. - Strong absolute effects, with high technical and clinical success rates. Relative effects not in one direction, supporting the statement that considers EGBD as an alternative to PTGBD - No variability in information - Costs and resources are not considered   Strong   - High quality evidence: one RCT (Itoi T et al. 2015) with 73 patients and one meta-analysis of studies within a guideline (Mori Y et al. 2018). - Strong and comparable absolute effect, with high technical and clinical success rates for both options - No variability of information - Costs and resources are not considered, but marginal interest |
| **Which is the role of endoscopic transmural ultrasound-guided gallbladder drainage (EUS-GBD) in patients with ACC who are not suitable for surgery?**  6.8 EUS-GBD with lumen-apposing self-expandable metal stents (LAMSs) should be preferred to ETGBD, if performed by skilled endoscopists.  6.9 If a EUG-GBD is performed using metal stents, we recommend their removal within 4 weeks, in order to avoid food impaction with subsequent high risk of recurrence of ACC. | Moderate  Low | Strong   - Moderate quality evidence for the use of LAMSs: one systematic review and meta-analysis of 23 studies (Khan MA et al. 2017) suggests LAMSs is safe and effective. This statement is supported by retrospective reviews (Dollhopf et al. 2017, Irani S et al. 2015), with weak study designs with a sample size <300. - No variability of information - High technical and clinical success rates and low incidence of adverse events suggests strong absolute effects; however, the small sample size and poor study designs of the retrospective studies can lead to potentially significant effects and bias - Costs and resources are not considered   Weak   - Low quality evidence: one retrospective study of 12 patients (Kamata K et al. 2017), one prospective study of 30 patients (Irani S et al. 2017) and one case series of 13 patients (de la Serna-Higuera et al. 2013), with weak study designs and small sample sizes - Relative effects not in the same direction, as the case series suggests leaving metal stents (LAMSs) for up to three months may have beneficial effects - Costs and resources are not considered |
| **Which is the optimal antibiotic treatment for patients with uncomplicated ACC?**  7.1 In uncomplicated ACC, we recommend against the routine use of postoperative antibiotics when the focus of infection is controlled by cholecystectomy. | High | Strong   - High quality evidence:one RCT of 417 patients, strong study design and good sample size (Regimbeau JM et al. 2014) - Even though only one study is presented to support the statement and there is no evidence on variability of information, the large overlap of CI and the absence of statistical differences between the groups strongly supports the statement. - Costs and resources not stated, but marginal interest |
| **Which is the optimal antibiotic treatment for patients with complicated ACC?**  7.2 In complicated ACC, we recommend prescribing the antimicrobial regimen based on the presumed pathogens involved and the risk factors for major resistance patterns. | High | Strong   - High quality evidence: two multicentre prospective observational studies of 278 (Sartelli et al. 2014) and 289 (Sartelli et al. 2012) patients suggest the potential types of microrganism and indicate the need of targeted antimicrobial regimen depending on the presumed pathogen. Strong study design overall good sample size. Two prospective observational studies of 62 (Dhalluin-Venier et al. 2008) and 180 (Richè et al. 2009) patients, two expert consensuses (Dellinger et al. 2008, Singer and Deutschman et al. 2016) and one review article (Pea et al. 2009) support the statement - High patient importance, as different regimens directly influences prognosis |
| **Which is the role of microbiological cultures and sensitivities in patients with ACC?**  7.3 In patients with complicated ACC and patients at high risk for antimicrobial resistance, we recommend adapting the targeted antibiotic regime on the results of microbiological analysis, ensuring adequate antimicrobial coverage | Moderate | Weak   - Moderate quality evidence: two prospective observational study of 372 (Csendes et al. 1994) and 467 (Csendes et al. Arch. Surg 1996) patients, three retrospective observational study of 208 (Csendes et al. 1996), 1394 (Chang et al. 2002) and 125 (Salvador et al. 2011) patients. Good sample size, however weak study designs as all the studies are observational - Indirectness of evidence as all evidence simply provides positive culture rates. |

## Appendix 2. High-risk patients with ACC and patients with ACC who are not suitable for surgery

One of the main efforts of the WSES was to develop an evidence-based identification of homogeneous cohorts of patients presenting with ACC, in order to develop appropriate treatment recommendations. In the CHOCOLATE study^11^, high-risk patient are defined by an APACHE II score of 7-14; although surgery probably represents the preferable treatment option in this group of patients, there are clinical conditions which are not amenable for categorization by a physiologic score, such as the APACHE II score.

Within the category of high-risk patients, the recent RCT by Teoh et al. could indirectly identify a group of patients who were poor candidates for surgery based on their baseline characteristics (presenting with one of the following age > 80 years, ASA grade ≥3 or an age-adjusted Charlson Comorbidity Score > 5 and/or a Karnofsky score <50) and who have fairly good outcomes with EUS-GBD, when compared to PTGBD. The CHOCOLATE study had previously reported that urgent laparoscopic cholecystectomy was superior to PTGBD in a group of high-risk patients, who presented with deranged physiology (APACHE II score > 7 - ‘high risk for surgery’ in the current guidelines). The populations of the two studies, although in part overlapping, are likely to be different; therefore, no consideration on the role of EUS-GBD in ‘high risk patients’ can be obtained from current evidence. According to the Authors the patients with these characteristics could be considered unsuitable for surgery. However, evidence on this definition is not consistent.

Consequently, the WSES proposes the definition of two groups of patients:

1. *High-risk patients with ACC*: patients presenting with an APACHE score of 7-14, who are therefore at high-risk for mortality and morbidity, also according to local guidelines, local use of clinical scores and local clinical agreement. The correction of the physiologic status of the patient may allow for a safe surgical treatment of ACC.
2. *Patients with ACC who are not suitable for surgery*: patients who, according to the surgeon’s clinical judgement, are not fit for surgery due to clinical conditions which are not classifiable with the use of a score and which make surgery unsafe or impossible.

The agreement for both reached 100% consensus among experts in the online survey.
